# Targeting of cytosolic phospholipase A_2_α impedes cell cycle re-entry of quiescent prostate cancer cells

**DOI:** 10.18632/oncotarget.5277

**Published:** 2015-09-24

**Authors:** Mu Yao, Chanlu Xie, Mei-Yee Kiang, Ying Teng, David Harman, Jessamy Tiffen, Qian Wang, Paul Sved, Shisan Bao, Paul Witting, Jeff Holst, Qihan Dong

**Affiliations:** ^1^ Department of Endocrinology, Royal Prince Alfred Hospital, Camperdown, NSW 2050, Australia; ^2^ Central Clinical School and Bosch Institute, The University of Sydney, Sydney, NSW 2006, Australia; ^3^ School of Biomedical and Health Sciences, University of Western Sydney, Parramatta, NSW 2751, Australia; ^4^ Molecular Medicine Research Group, University of Western Sydney, Parramatta, NSW 2751, Australia; ^5^ Origins of Cancer Laboratory, Centenary Institute, Camperdown, NSW 2050, Australia; ^6^ Sydney Medical School, The University of Sydney, Sydney, NSW 2006, Australia; ^7^ Department of Urology, Royal Prince Alfred Hospital, Camperdown, NSW 2050, Australia; ^8^ Department of Pathology, The University of Sydney, Sydney, NSW 2006, Australia

**Keywords:** prostate cancer, phospholipase A2α, cell cycle re-entry, p27, Skp2

## Abstract

Cell cycle re-entry of quiescent cancer cells has been proposed to be involved in cancer progression and recurrence. Cytosolic phospholipase A_2_α (cPLA_2_α) is an enzyme that hydrolyzes membrane glycerophospholipids to release arachidonic acid and lysophospholipids that are implicated in cancer cell proliferation. The aim of this study was to determine the role of cPLA_2_α in cell cycle re-entry of quiescent prostate cancer cells. When PC-3 and LNCaP cells were rendered to a quiescent state, the active form of cPLA_2_α with a phosphorylation at Ser^505^ was lower compared to their proliferating state. Conversely, the phospho-cPLA_2_α levels were resurgent during the induction of cell cycle re-entry. Pharmacological inhibition of cPLA_2_α with Efipladib upon induction of cell cycle re-entry inhibited the re-entry process, as manifested by refrained DNA synthesis, persistent high proportion of cells in G_0_/G_1_ and low percentage of cells in S and G_2_/M phases, together with a stagnant recovery of Ki-67 expression. Simultaneously, Efipladib prohibited the emergence of Skp2 while maintained p27 at a high level in the nuclear compartment during cell cycle re-entry. Inhibition of cPLA_2_α also prevented an accumulation of cyclin D1/CDK4, cyclin E/CDK2, phospho-pRb, pre-replicative complex proteins CDC6, MCM7, ORC6 and DNA synthesis-related protein PCNA during induction of cell cycle re-entry. Moreover, a pre-treatment of the prostate cancer cells with Efipladib during induction of cell cycle re-entry subsequently compromised their tumorigenic capacity *in vivo*. Hence, cPLA_2_α plays an important role in cell cycle re-entry by quiescent prostate cancer cells.

## INTRODUCTION

Prostate cancer is a common malignancy [1] and leading cause of cancer-related mortality in men [2]. Clinical studies have demonstrated that the progression and recurrence of the cancer following initial treatment account for the mortality amongst the patients [3–5]. Therefore, an understanding of the mechanisms impinging on the progression and recurrence of the disease is pivotal in the control of the cancer-related mortality.

The presence of quiescent cancer cells has been documented in many types of tumors [6]. These quiescent cells are defined as being Ki-67 negative and in a reversible arrest state at G_0_ [7]. In prostate cancer, the percentage of Ki-67 positive cancer cells is low in low grade and low volume disease [8]. However, an increase in Ki-67 positive cancer cells is correlated with disease severity [9] and is associated with the risk of disease progression [10]. These observations suggest that a cell cycle re-entry of previously quiescent cancer cells has an adverse impact on clinical outcome. However, our knowledge of the biochemical signals required for quiescent prostate cancer cells to re-enter the cell cycle is limited. As a result, there is no effective therapeutic strategy to control the re-entry process of these quiescent cancer cells.

Phospholipase A_2_ (PLA_2_) is a family of enzymes that catalyze the hydrolysis of membrane phospholipids at the *sn*-2 position to release bioactive lipids [11]. In this regard, cytosolic PLA_2_α (cPLA_2_α) preferentially cleaves arachidonic acid (AA) from phospholipids. The free AA is then metabolized by a raft of downstream enzymes to produce eicosanoids, while the other enzymatic product lysophospholipid is converted to the platelet activating factor (PAF) by lyso-PAF acetyltransferase [12]. It is important to note that phosphorylation of cPLA_2_α at Ser^505^ can enhance the AA releasing property of this enzyme [13].

Both laboratory and clinical studies have suggested that AA and PAF contribute to prostate cancer progression and recurrence. Using a xenograft model of prostate cancer, McEntee et al. demonstrated that among the diets supplemented with different fatty acids, high dietary AA had a short time interval between the nadir of tumor regression and subsequent relapse of the regressed tumor following androgen ablation. In parallel, the reduction in time to relapse had the highest correlation with increased levels of AA in the tumor [14]. In another study Kelavkar et al. showed that the diet supplemented with linoleic acid, a precursor to AA, promoted tumor recurrence following surgical removal of xenograft of prostate cancer [15]. *In vitro*, AA and its metabolites were found to promote proliferation of human prostate cancer cells [16]. Interestingly, the rates of eicosanoid synthesis in the presence of spiked AA were enhanced in prostate cancer tissues compared to those in the benign tissues, suggesting an increased utilization of AA in the cancerous tissues [17]. The rate of rise in the prostate specific antigen level following prostatectomy or radiotherapy was slowed in patients treated with inhibitors of eicosanoid-producing enzymes in two independent clinical studies [18, 19]. Likewise, PAF antagonists have been shown to inhibit proliferation and induce apoptosis in prostate cancer cells [20, 21].

Since cPLA_2_α action is a key step in determining the bio-availability of AA and PAF [22], we hypothesize that inhibition of cPLA_2_α would prevent quiescent prostate cancer cells from re-entering the cell cycle. In this study, using two experimental models of quiescence in prostate cancer cells, we determined the effect of an indole cPLA_2_α inhibitor Efipladib [23] on cell cycle re-entry and examined the potential mechanism underlying cPLA_2_α action on the cell cycle re-entry.

## RESULTS

### Experimental quiescence rendered by contact inhibition in PC-3 cells

**Figure 1:**
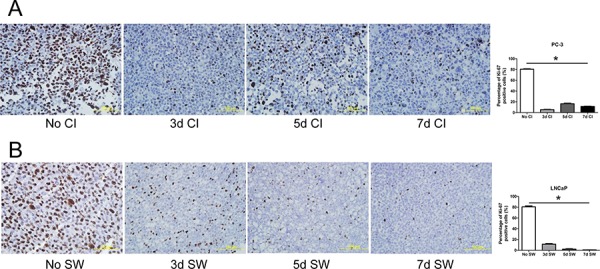
Induction of prostate cancer cells to quiescence **A.** PC-3 cells were maintained in a confluent state in T75 flasks for indicated time intervals. Thereafter, the cells were collected for analysis of Ki-67 by immunocytochemical staining. No CI: no contact inhibition; 3dCI: contact inhibition for 3 days; 5dCI: contact inhibition for 5 days; 7dCI: contact inhibition for 7 days. Histogram illustrates the percentage of Ki-67 positive cells. Data represents mean ± SD from three experiments. * Statistical significance compared to no contact inhibition (*p* < 0.01). **B.** LNCaP cells were serum-deprived in T75 flasks for indicated period of time. They were then collected for analysis of Ki-67 by immunocytochemical staining. No SW: no serum withdrawal; 3d SW: serum withdrawal for 3 days; 5d SW: serum withdrawal for 5 days; 7d SW: serum withdrawal for 7 days. Histogram represents the percentage of Ki-67 positive cells. Data represents mean ± SD from three experiments. * Different compared to no serum withdrawal (*p* < 0.01).

To establish quiescence by contact inhibition, PC-3 cells were maintained at 100% confluency (referred to as confluence from here onwards) for different time intervals. We employed flow cytometric analysis and immunocytochemical staining of Ki-67, a marker of proliferating cells [24, 25], to monitor the induction of quiescence. Flow cytometric analysis revealed that confluence caused no significant change in cell viability as reflected by the percentage of sub-G_1_ population. However, there was a significant increase in G_0_/G_1_ and a decrease in S and G_2_/M populations 3 days after confluence, compared to non-confluent cultures (Table 1A). In parallel, immunocytochemical staining demonstrated a marked decrease in the percentage of Ki-67 positive cells (Figure 1A). Intriguingly, there was an increment in Ki-67 positivity in the cultures with 5 and 7 day confluence compared to 3 day confluent culture (Figure 1A). In addition, there was a moderate reverse change in cell cycle phase distribution 5 and 7 days after confluence (Table 1A). Therefore, 3 day confluence was chosen to synchronize PC-3 cells into quiescence.

**Table 1A:** Analysis of quiescent state in PC-3 by flow cytometry

	Sub-G_1_	G_0_/G_1_	S	G_2_/M
No confluence	0.18 ± 0.01	51.4 ± 0.3	16.4 ± 1.2	31.9 ± 1.7
3 day confluence	0.29 ± 0.15	82.7 ± 2.8*	2.1 ± 1.7*	14.7 ± 0.8*
5 day confluence	0.16 ± 0.05	75.7 ± 0.2*	3.2 ± 0.2*	20.9 ± 0.3*
7 day confluence	0.07 ± 0.03	78.0 ± 0.4*	2.7 ± 0.0*	19.1 ± 0.4*

PC-3 cells were maintained at confluency in T25 flasks for the indicated time periods and then analyzed for cell cycle phase distribution. Data represent mean ± SD from three experiments.

* Different compared to no confluence (*p* < 0.05).

### Experimental quiescence rendered by serum withdrawal in LNCaP cells

To establish cell quiescence by serum withdrawal, LNCaP cells were serum-deprived for various time intervals. There was a significant increase in G_0_/G_1_ population and a decrease in S and G_2_/M populations following serum withdrawal for 3, 5 and 7 days, compared to the cells cultured in the presence of serum (Table 1B). Although it is notable that there was an increasing frequency of sub-G_1_ over the time of serum withdrawal, the extent to which cell viability became compromised was negligible (<3%). Concomitantly, a substantial reduction in Ki-67 positivity was observed after 3 to 5 day serum withdrawal (Figure 1B). There was a further decrease in the percentage of cells expressing Ki-67 after 7 day serum deprivation (Figure 1B). Therefore, 7 day serum withdrawal was employed in all further studies to render quiescence in LNCaP cells.

**Table 1B:** Analysis of quiescent state in LNCaP cells by flow cytometry

	Sub-G_1_	G_0_/G_1_	S	G_2_/M
No serum withdrawal	0.15 ± 0.05	59.1 ± 0.2	23.6 ± 0.6	17.0 ± 0.3
3 day serum withdrawal	1.05 ± 0.15*	86.4 ± 0.3*	4.7 ± 0.1*	7.4 ± 0.3*
5 day serum withdrawal	1.70 ± 0.21*	89.1 ± 0.3*	3.7 ± 0.2*	5.3 ± 0.2*
7 day serum withdrawal	2.34 ± 0.16*	89.8 ± 0.1*	3.28 ± 0.2*	4.5 ± 0.1*

LNCaP cells were serum-deprived in T25 flasks for indicated period of time and subsequently analyzed for cell cycle phase distribution. Data represent mean ± SD from three experiments.

* Different compared to no serum withdrawal (*p* < 0.05).

### Modulation of phosphorylation on cPLA_2_α during transition of cell cycle status

**Figure 2:**
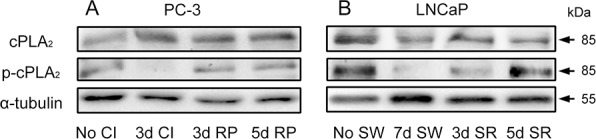
Modulation of phosphorylation on cPLA_2_α during transition of cell cycle status **A.** PC-3 cells were rendered to quiescent status by 3 day contact inhibition and then induced to re-enter the cell cycle by re-plating them at a low density (1:6 dilution) in 6-well plates. **B.** LNCaP cells were made quiescent by 7 day serum withdrawal and then induced to re-enter the cell cycle by re-plating them in the presence of serum in 6-well plates. The cells in both A and B were then harvested at indicated time intervals for immunoblot analysis of both cPLA_2_α and phosphorylated cPLA_2_α. No CI: no contact inhibition; 3d CI: contact inhibition for 3 days; 3d RP: re-plate cells for 3 days; 5d RP: re-plate cells for 5 days. No SW: no serum withdrawal; 7d SW: serum withdrawal for 7 days; 3d SR: serum replenished for 3 days; 5d SR: serum replenished for 5 days.

To determine whether there was an association between cPLA_2_α expression or its phosphorylation and cell cycle state in prostate cancer cells, both total cPLA_2_α and phosphorylated cPLA_2_α (p-cPLA_2_α) at Ser^505^ were analyzed by immunoblotting. While total cPLA_2_α levels were largely unchanged in quiescent prostate cancer cells compared to the non-synchronized proliferative cultures, levels of phosphorylated cPLA_2_α diminished. However, decreased phosphorylation on cPLA_2_α was restored to the levels comparable to those in non-synchronized cultures 3 days in PC-3 cells and 5 days in LNCaP cells following an induction of cell cycle re-entry (Figure 2 and Supplementary Figure 1). The cell cycle status was confirmed by immunocytochemical staining of Ki-67 (Supplementary Figure 2). These results suggest that cPLA_2_α may play a role in the cell cycle re-entry by quiescent prostate cancer cells.

### Pharmacological inhibition of cPLA_2_α blocks cell cycle re-entry of quiescent prostate cancer cells

**Figure 3:**
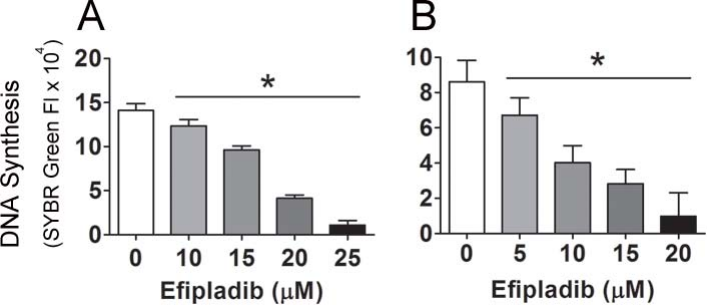
Blockade of the cell cycle re-entry of quiescent prostate cancer cells by cPLA_2_α inhibition as measured for DNA content Quiescent PC-3 in **panel A** and LNCaP cells in **panel B** were allowed to re-enter cell cycle in 96-well plates and unused cells were stored at −80°C as baselines. Different doses of Efipladib or DMSO as vehicle control were administered upon re-plating the cells. Three days later, the treated cells in the plates, together with the cells used as baselines, were subjected to lysis buffer containing SYBRGreen and their DNA contents were measured. The net increase in DNA content in the experimental cells was estimated by subtracting baseline value from the value of the treated cells and the results are expressed as mean ± SD. * Different between DMSO and Efipladib (*p* < 0.05).

To determine the role of cPLA_2_α in cell cycle re-entry by quiescent prostate cancer cells, both quiescent PC-3 and LNCaP cells were treated with Efipladib, a selective and potent inhibitor of cPLA_2_α [23], upon an induction of cell cycle re-entry. As one of the hallmarks of cell cycle re-entry is a re-synthesis of DNA [26], we first employed a SYBRGreen assay to monitor this process during induction of cell cycle re-entry. Treatment with Efipladib for 3 days during induction of cell cycle re-entry significantly inhibited DNA synthesis in both quiescent PC-3 and LNCaP cells in a dose-dependent manner (Figure 3).

**Figure 4:**
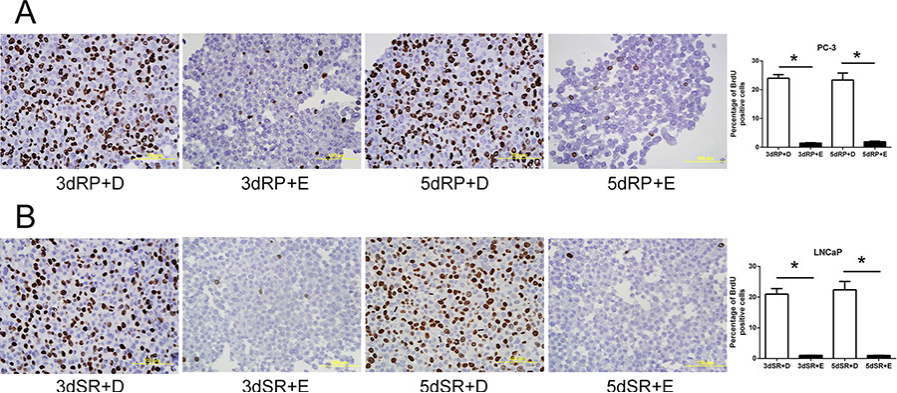
Blockade of the cell cycle re-entry of quiescent prostate cancer cells by cPLA_2_α inhibition as measured for BrdU incorporation Both quiescent PC-3 in **panel A** and LNCaP cells in **panel B** were permitted to re-enter cell cycle and either 25 μM Efipladib or DMSO was administered to the cells. Three or five days after treatment, the cells were harvested for analysis of incorporated BrdU by immunocytochemical staining. The histograms illustrate the percentage of BrdU positive cells and data represent mean ± SD from three experiments. * Different to the vehicle control at each corresponding time interval (*p* < 0.01). 3dRP+D: treatment of re-plated (RP) cells with DMSO (D) for 3 days; 3dRP+E: treatment of re-plated cells with Efipladib (E) for 3 days; 5dRP+D: treatment of re-plated cells with DMSO for 5 days; 5dRP+E: treatment of re-plated cells with Efipladib for 5 days. 3dSR+D: treatment of serum-replenished (SR) cells with DMSO for 3 days; 3dSR+E: treatment of serum-replenished cells with Efipladib for 3 days; 5dSR+D: treatment of serum-replenished cells with DMSO for 5 days; 5dSR+E: treatment of serum-replenished cells with Efipladib for 5 days.

**Figure 5:**
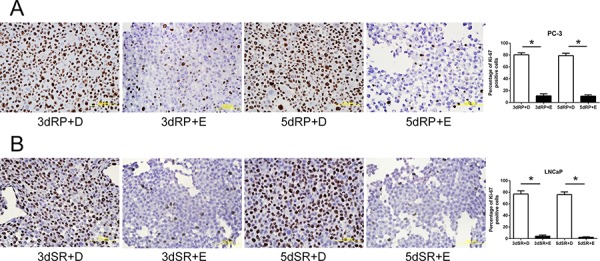
Blockade of the cell cycle re-entry of quiescent prostate cancer cells by cPLA_2_α inhibition as measured for Ki-67 positivity Quiescent PC-3 in **panel A** and LNCaP cells in **panel B** were induced to re-enter cell cycle and treated with either 25 μM Efipladib or DMSO. Three or five days after treatment, the cells were harvested for analysis of Ki-67 by immunocytochemical staining. The histograms show the percentage of Ki-67 positive cells and data represent mean ± SD from three experiments. * Different to the vehicle control at each corresponding time interval (*p* < 0.01). 3dRP+D: treatment of re-plated (RP) cells with DMSO (D) for 3 days; 3dRP+E: treatment of re-plated cells with Efipladib (E) for 3 days; 5dRP+D: treatment of re-plated cells with DMSO for 5 days; 5dRP+E: treatment of re-plated cells with Efipladib for 5 days. 3dSR+D: treatment of serum-replenished (SR) cells with DMSO for 3 days; 3dSR+E: treatment of serum-replenished cells with Efipladib for 3 days; 5dSR+D: treatment of serum-replenished cells with DMSO for 5 days; 5dSR+E: treatment of serum-replenished cells with Efipladib for 5 days.

To confirm the inhibitory effect of cPLA_2_α on the DNA synthesis, Efipladib was next administered respectively to PC-3 and LNCaP cells at 25 μM and 20 μM when the cells were induced to re-enter the cell cycle. BrdU incorporation was measured 3 or 5 days thereafter. Notably, BrdU incorporation was consistently suppressed in Efipladib-treated cells compared to vehicle-treated controls at both time intervals tested (Figure 4). Simultaneously, flow cytometric analysis illustrated that cell cycle re-entry was hindered in both cancer cell lines treated with Efipladib for 3 or 5 days in comparison with the control, as manifested by a higher percentage of cells in G_0_/G_1_ and a lower proportion of cells in S and G_2_/M (Tables 2A and 2B). In line with these observations, Efipladib inhibited the recovery of Ki-67 positivity in both cell lines at the intervals of interrogation as judged by immunocytochemical analysis (Figure 5). Taken together, these results indicate that cPLA_2_α is required for cell cycle re-entry by quiescent prostates cancer cells.

**Table 2A:** The inhibitory effect of Efipladib on cell cycle re-entry in PC-3 cells

	Sub-G_1_	G_0_/G_1_	S	G_2_/M
3 day RP + DMSO	0.13 ± 0.05	59.0 ± 2.1	10.9 ± 0.3	28.9 ± 1.5
3 day RP + Efipladib	0.06 ± 0.03	80.0 ± 1.5*	3.8 ± 0.3*	15.8 ± 1.1*
5 day RP + DMSO	0.08 ± 0.04	55.5 ± 0.7	12.9 ± 0.3	30.1 ± 0.7
5 day RP + Efipladib	0.06 ± 0.02	75.9 ± 1.5*	5.8 ± 0.2*	17.8 ± 1.1*

Quiescent PC-3 cells were allowed to re-enter cell cycle by re-plating them at a low density in T25 flasks. Either 25 μM Efipladib or DMSO was introduced upon re-plating the cells. Three or five days later, the cells were harvested and analyzed by flow cytometry for cell cycle phase distribution. RP: re-plating. Data represent mean ± SD from three experiments.

* Different to the vehicle control at each time interval (*p* < 0.05).

**Table 2B:** The inhibitory effect of Efipladib on cell cycle re-entry in LNCaP cells

	Sub-G_1_	G_0_/G_1_	S	G_2_/M
3 day SR + DMSO	0.23 ± 0.05	70.6 ± 0.6	14.1 ± 0.5	15.1 ± 0.25
3 day SR + Efipladib	0.20 ± 0.07	89.5 ± 1.6*	3.2 ± 0.1*	6.9 ± 0.5*
5 day SR + DMSO	0.18 ± 0.06	67.8 ± 1.5	17.5 ± 0.5	14.6 ± 0.3
5 day SR + Efipladib	0.26 ± 0.05	90.8 ± 1.6*	2.9 ± 0.2*	6.2 ± 0.7*

Quiescent LNCaP cells were permitted to re-enter cell cycle by re-plating them in the presence of serum in T25 flasks. Either 20 μM Efipladib or DMSO was administered upon serum restoration. Three or five days later, the cells were harvested and analyzed for cell cycle phase distribution. SR: serum restoration. Data represent mean ± SD from three experiments.

* Different to the vehicle control at each time interval (*p* < 0.05).

### Inhibition of cPLA_2_α suppresses the accumulation of cyclin/CDK and phosphorylation on Rb protein (pRb) during cell cycle re-entry

During cell cycle re-entry the level of cyclin D1 is known to increase [27]. Increased cyclin D1 together with CDK4 or CDK6 can phosphorylate pRb. Subsequently, cyclin E–CDK2 phosphorylates it at additional sites [28]. The phosphorylation of pRb results in its dissociation from E2F transcription factors, permitting them to initiate transcription of their target genes [29] and to facilitate entry into S phase [30].

**Figure 6:**
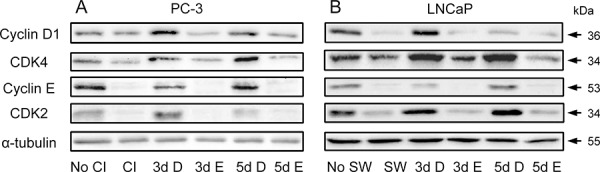
Modulation of cyclins and CDKs by Efipladib during cell cycle re-entry **A.** Quiescent PC-3 cells were allowed to re-enter cell cycle by re-plating them at a low density in 6-well plates. Either 25 μM Efipladib or DMSO was added upon re-plating the cells. Three or five days after treatment, the cells were harvested for immunoblot analysis. No CI: no contact inhibition; 3dCI: contact inhibition for 3 days; 3d D: treatment of re-plated cells with DMSO (D) for 3 days; 3d E: treatment of re-plated cells with Efipladib (E) for 3 days; 5d D: treatment of re-plated cells with DMSO (D) for 5 days; 5d E: treatment of re-plated cells with Efipladib (E) for 5 days. **B.** Quiescent LNCaP cells were allowed to re-enter cell cycle by re-plating them in the presence of serum in 6-well plates. Either 20 μM Efipladib or DMSO was administered upon serum restoration. Three or five days after treatment, the cells were harvested for immunoblot analysis. No SW: no serum withdrawal (SW); SW: serum withdrawal for 7 days; 3d D: treatment of serum-replenished cells with DMSO (D) for 3 days; 3d E: treatment of serum-replenished cells with Efipladib (E) for 3 days; 5d D: treatment of serum-replenished cells with DMSO (D) for 5 days; 5d E: treatment of serum-replenished cells with Efipladib (E) for 5 days. The results are representative of three experiments.

**Figure 7:**
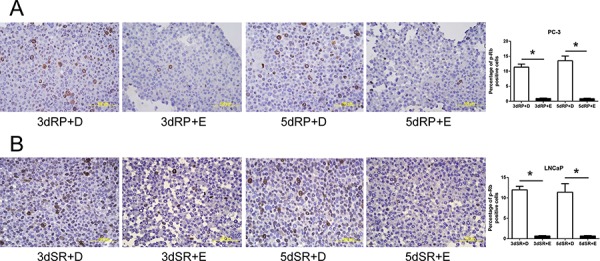
Inhibition of phosphorylation on pRb by Efipladib during cell cycle re-entry **A.** Quiescent PC-3 cells were allowed to re-enter cell cycle by re-plating them at a low density in T75 flasks. Either 25 μM Efipladib or DMSO was added upon re-plating the cells. Three or five days after treatment, the cells were harvested for analysis of phosphorylated pRb by immunocytochemical staining. **B.** Quiescent LNCaP cells were permitted to re-enter cell cycle by re-plating them in the presence of serum in T75 flasks. Either 20 μM Efipladib or DMSO was administered upon serum restoration. Three days or five days after treatment, the cells were collected for analysis of phosphorylation on pRb by immunocytochemical staining. The histograms in A and B show the percentage of cells stained for phospho-pRb (p-pRb). Data represent mean ± SD from three experiments. * Different to the vehicle control at each corresponding time interval (*p* < 0.01). 3dRP+D: treatment of re-plated (RP) cells with DMSO (D) for 3 days; 3dRP+E: treatment of re-plated cells with Efipladib (E) for 3 days; 5dRP+D: treatment of re-plated cells with DMSO for 5 days; 5dRP+E: treatment of re-plated cells with Efipladib for 5 days. 3dSR+D: treatment of serum-replenished (SR) cells with DMSO for 3 days; 3dSR+E: treatment of serum-replenished cells with Efipladib for 3 days; 5dSR+D: treatment of serum-replenished cells with DMSO for 5 days; 5dSR+E: treatment of serum-replenished cells with Efipladib for 5 days.

To determine if these cell cycle regulators were influenced by cPLA_2_α inhibition, PC-3 and LNCaP cells were treated with or without Efipladib for 3 or 5 days during cell cycle re-entry and their levels were subsequently measured. Immunoblot analysis illustrated that these regulators generally were down-regulated in the quiescent state compared to the proliferative status. During the course of cell cycle re-entry, their levels were elevated at both time points. However, their re-accumulation was repressed in the presence of Efipladib during the induction of cell cycle re-entry (Figure 6 and Supplementary Figure 3). Consistently, the phosphorylation of pRb at Ser^807/811^, two of the residues that need to be phosphorylated for lifting its suppressive effect on E2F-induced gene transcription [28], was remarkably reduced by the administration of Efipladib when the quiescent cancer cells were permitted to re-enter cell cycle (Figure 7).

### Inhibition of cPLA_2_α prevents a recovery of the components involved in DNA synthesis during cell cycle re-entry

**Figure 8:**
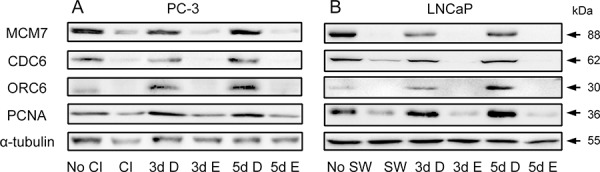
Modulation of Pre-RC proteins and PCNA by Efipladib during cell cycle re-entry **A.** Quiescent PC-3 cells were allowed to re-enter cell cycle by re-plating them at a low density in 6-well plates. Either 25 μM Efipladib or DMSO was added upon re-plating the cells. Three or five days after treatment, the cells were harvested for immunoblot analysis. No CI: no contact inhibition; 3d CI: contact inhibition for 3 days; 3d D: treatment of re-plated cells with DMSO (D) for 3 days; 3d E: treatment of re-plated cells with Efipladib (E) for 3 days; 5d D: treatment of re-plated cells with DMSO (D) for 5 days; 5d E: treatment of re-plated cells with Efipladib (E) for 5 days. **B.** Quiescent LNCaP cells were permitted to re-enter cell cycle by re-plating them in the presence of serum in 6-well plates. Either 20 μM Efipladib or DMSO was administered upon serum restoration. Three or five days after treatment, the cells were collected for immunoblot analysis. No SW: no serum withdrawal (SW); 7d SW: serum withdrawal for 7 days; 3d D: treatment of serum-replenished cells with DMSO (D) for 3 days; 3d E: treatment of serum-replenished cells with Efipladib (E) for 3 days; 5dSR+D: treatment of serum-replenished cells with DMSO (D) for 5 days; 5d E: treatment of serum-replenished cells with Efipladib (E) for 5 days. The results are representative of three experiments.

An assembly of pre-replication complex (pre-RC) on DNA, termed as licensing, is a pre-requisite for DNA replication. Licensing begins at replication origin in early G_1_ with sequential loading of ORCs, CDC6, Cdt1 and putative helicase MCMs [31]. It has been shown that a loss of proliferative capacity in the quiescent state is achieved partly through down-regulation of CDC6 and MCMs [32]. Conversely, G_1_ traverse is accelerated when CDC6 is introduced to G_1_ nuclei [26]. In addition, PCNA is also involved in DNA synthesis by acting as a processivity factor of DNA polymerase δ [33]. Therefore, we determined if cPLA_2_α inhibition had any effect on the levels of these components that are involved in DNA synthesis. During the induction of cell cycle re-entry, pre-RC components MCM7, CDC6 and ORC6 together with PCNA were accumulated in both PC-3 and LNCaP cells. However, their accumulations were suppressed when Efipladib was introduced to the cancer cells during the induction of cell cycle re-entry (Figure 8 and Supplementary Figure 4).

### Inhibition of cPLA_2_α retains p27 while preventing recovery of Skp2 during cell cycle re-entry

**Figure 9:**
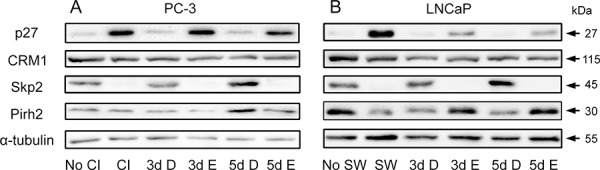
Modulation of p27 and its regulators by Efipladib during cell cycle re-entry **A.** Quiescent PC-3 cells were induced to re-enter the cell cycle by re-plating them at a low density in 6-well plates. Either 25 μM Efipladib or DMSO was added upon re-plating the cells. Three or five days after treatment, the cells were harvested for immunoblot analysis. No CI: no contact inhibition; 3d CI: contact inhibition for 3 days; 3d D: treatment of re-plated (RP) cells with DMSO (D) for 3 days; 3d E: treatment of re-plated cells with Efipladib (E) for 3 days; 5d D: treatment of re-plated cells with DMSO (D) for 5 days; 5d E: treatment of re-plated cells with Efipladib (E) for 5 days. **B.** Quiescent LNCaP cells were allowed to re-enter cell cycle by re-plating them in the presence of serum in 6-well plates. Either 20 μM Efipladib or DMSO was administered upon serum restoration. Three or five days after treatment, the cells were collected for immunoblot analysis. No SW: no serum withdrawal; 7d SW: serum withdrawal for 7 days; 3d D: treatment of serum-replenished cells with DMSO (D) for 3 days; 3d E: treatment of serum-replenished cells with Efipladib (E) for 3 days; 5d D: treatment of serum-replenished cells with DMSO (D) for 5 days; 5d E: treatment of serum-replenished cells with Efipladib (E) for 5 days. The results are representative of three experiments.

A high level of cyclin-dependent kinase (CDK) inhibitor p27 is found in quiescent cells and its elevated level is required for the maintenance of quiescent state [34]. We therefore determined the expression of p27 and its regulatory proteins CRM1, Skp2 and Pirh2 during cell cycle re-entry by quiescent prostate cancer cells. Quiescent PC-3 and LNCaP cells were allowed to re-enter cell cycle with or without Efipladib for 3 or 5 days. Immunoblot analysis showed that in the absence of Efipladib p27 levels diminished during cell cycle re-entry in both cancer cell lines compared to those seen in quiescent status. In contrast, cells exposed to Efipladib maintained higher levels of p27 (Figure 9 and Supplementary Figure 5). Consistently, the percentage of cells with nuclear p27 was also higher when cell cycle re-entry was induced in the presence of Efipladib (Supplementary Figures 6 and 7).

To determine if the proteins that promote p27 turnover were affected by cPLA_2_α, immunoblot analysis of CRM1, Skp2 and Pirh2 was conducted. While CRM1 levels were not largely affected by the treatment, the restoration of Skp2 from low levels seen in quiescent state was evidently suppressed during cell cycle re-entry when Efipladib was administered. Interestingly, there was a divergence in the change of Pirh2 between PC-3 and LNCaP cells in response to the treatment. Whereas Efipladib had no obvious effect on Pirh2 levels in PC-3 cells, the treatment increased the protein levels in LNCaP cells at day 3 and day 5 following induction of cell cycle re-entry (Figure 9 and Supplementary Figure 5).

### The impact of cPLA_2_α inhibition imposed during cell cycle re-entry on tumorigenic capacity of prostate cancer cells

**Figure 10:**
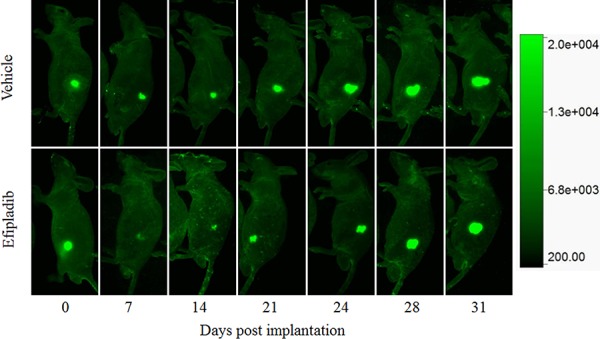
The impact on tumorigenic capacity following treatment with Efipladib during cell cycle re-entry PC-3 cells expressing GFP were rendered to quiescence and then induced to re-enter the cell cycle by re-plating them in T75 flasks in the presence or absence of Efipladib for 5 days. Thereafter, both vehicle control and treated cells were collected and implanted to athymic nude mice subcutaneously. The amount of GFP expressed in the implanted cells was measured to monitor their growth potential under *in vivo* condition. The images are the representatives of GFP-expressing tumor at different time points.

**Figure 11:**
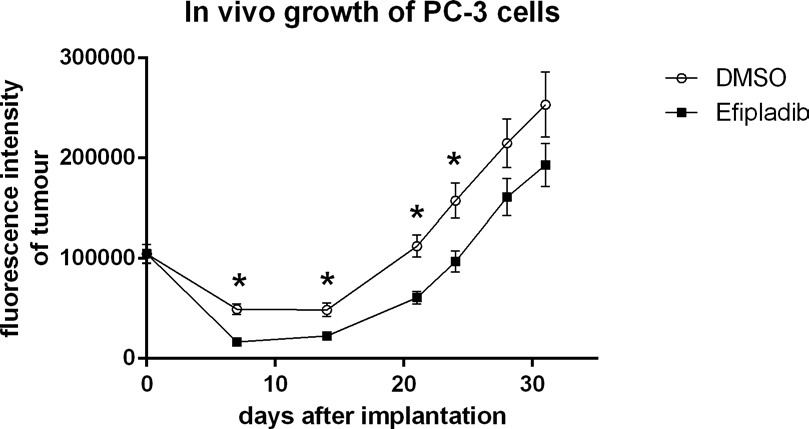
The impact on tumorigenic capacity after Efipladib treatment during cell cycle re-entry PC-3 cells expressing GFP were treated and implanted to recipient mice as described in Figure 10 legend. The graph illustrates the trend of the tumor growth over the monitoring course. The data represent mean ± SD. * Different to vehicle control (*p* < 0.01).

As cPLA_2_α inhibition persistently blocked cell cycle re-entry of quiescent prostate cancer, we reasoned that the treatment could have a negative impact on their tumorigenic capacity under *in vivo* conditions. To corroborate the proposition, quiescent PC-3 cells expressing GFP were re-plated to induce cell cycle re-entry in the presence or absence of Efipladib for 5 days. Thereafter, the cells were implanted to athymic nude mice subcutaneously and the amount of GFP expressed in the implanted cells was measured to monitor their growth potential. Seven days after cell implantation, the amount of GFP in the treated cohort decreased by ∼84% compared to its baseline at day 0. This amplitude of decline was significantly higher than that observed in the vehicle control where the reduction was ∼51% (*P* < 0.01). The GFP amount in both cohorts then gradually increased and approximated baseline levels after 21 days in the control or 24 days in the treatment group. Thereafter, their GFP amount continued to increase, and subsequently almost doubled the baseline levels after 28 days in the control or 31 days in the treatment group. The difference of GFP amounts between the two cohorts was persistently significant until 28 days after cell implantation (Figures 10 and 11). Therefore, the adopted mode of cPLA_2_α inhibition imposed upon quiescent prostate cancer cells compromised their tumorigenic capacity in an anchorage-independent environment.

## DISCUSSION

Evidence has emerged that cPLA_2_α is implicated in cancer development [35]. Previously we showed that over-expression of cPLA_2_α enhances its activity and liberation of arachidonic acid in cancer cells, which is accompanied by an increase in AKT signaling and cell proliferation [36, 37]. Conversely, inhibition of cPLA_2_α in cancer cells by either genetic silencing or pharmacological blocking suppressed its activity and release of arachidonic acid, which is concurrent with a reduction in the AKT signaling and cell proliferation [36–38]. Furthermore, a pharmacological blockade with cPLA_2_α inhibitor reduces phosphorylation of cPLA_2_α [37], which is required for the enhancement of its activity in hydrolysis of phospholipid to liberate arachidonic acid [13].

To test the hypothesis that blocking cPLA_2_α with Efipladib is capable of preventing quiescent prostate cancer cells from re-entering the cell cycle, we first established two models of experimental quiescence by contact inhibition in PC-3 cells and serum deprivation in LNCaP cells. Although both methods have been used to synchronize cells into quiescent state in previous studies [32, 39, 40], the timeframe required for enriching quiescent cells by either contact inhibition or serum deprivation has not been well defined. Time course experiments revealed that 3 day confluence effectively rendered PC-3 cells into a quiescent state compared to other time intervals. In LNCaP cells, quiescence was maximally achieved by 7 days of serum deprivation compared to other time points. It is noteworthy that both experimental models of quiescence had negligible impact on cell viability. These results will be instrumental for future studies involving quiescent cells as an experimental model. We then determined the efficacy of cPLA_2_α blockade with Efipladib in inhibition of cell cycle re-entry by quiescent prostate cancer cells synchronized by two independent methods. Treatment with Efipladib prevented the quiescent cancer cells from re-entering cell cycle as manifested by repressed resumption of DNA synthesis, restrained recovery of Ki-67 positivity and persistent pattern of cell cycle phase that was characterized by quiescent cancer cells.

It is noteworthy that over-expression of cyclin D1 and CDK4 could promote cell cycle re-entry without serum stimulation [41]. A higher level of cyclin D1 was found in metastasis of androgen-independent prostate cancer [42]. Over-expression of cyclin D1 and CDK4 were evident in the majority of human intestinal adenoma of familial adenomatous polyposis [43]. Equally significant, an over-expression of cyclin E has been shown to be associated with a poor prognosis in the patients with breast cancer [44], and down-regulation of cyclin E by siRNA suppressed tumor growth in a xenograft model of breast cancer [45]. In addition, cyclin E–CDK2 complex is able to phosphorylate p27 at Thr^187^ [46], which is required for its ubiquitination by Skp2-containing SCF complex and subsequent degradation by proteasome degradation [47]. Furthermore, cyclins and CDKs can bind to p27 and this binding promotes its turnover [39]. In comparison with quiescent status of prostate cancer cells, we noticed an accumulation in cyclin D1, CDK4, cyclin E and CDK2 together with the expected reappearance of phosphorylated pRb when the quiescent cancer cells were induced to re-enter the cell cycle. However, these changes associated with cell cycle re-entry were either abolished or reduced in the presence of Efipladib, which is consistent with the observation that the cell cycle re-entry was blocked by cPLA_2_α inhibition.

DNA replication is a hallmark in cell cycle re-entry [26]. The licensing factors together with other regulators including PCNA are essential in the initiation of DNA replication. Intriguingly, cyclin E has been reported to be an additional licensing factor. In the absence of cyclin E, MCMs fails to be loaded onto chromatin, and cyclin E-null mouse fibroblasts are unable to re-enter cell cycle from quiescent state [48, 49]. Over-expression of licensing proteins including ORCs, CDC6 and MCMs have been found in certain cancers and can be potentially used as markers for certain malignancies [50]. Likewise, PCNA level was found to be higher in prostate cancer than those in high-grade prostatic intraepithelial neoplasia and benign prostatic epithelium [51]. Furthermore, the expression of PCNA is significantly correlated with Gleason score and vascular invasion in prostate cancer [51]. Consistent with this line of evidence, we demonstrated that ORC6, DCD6, MCM7 together with PCNA and cyclin E were diminished in quiescent prostate cancer cells. By comparison, levels of these proteins were all up-regulated when the quiescent cancer cells were permitted to re-enter the cell cycle. This increase in protein levels was abrogated when Efipladib was introduced upon the induction of cell cycle re-entry.

As a member of CDK inhibitors, p27 possesses a broad spectrum of CDK-inhibitory activity [52]. The level of p27 is increased in quiescent cells, but is down-regulated when the quiescent cells are stimulated with growth factors [53]. Consistently, it negatively modulates cell cycle re-entry and therefore is required for maintenance of the quiescent state [34]. Not surprisingly, a low expression of p27 in cancer tissue is associated with poor clinical prognosis [54]. Recent studies have revealed that p27 can be regulated at the post-translational level by a variety of regulators including Skp2, Pirh2 and CRM1 [47, 55, 56].

As one of its important regulators, Skp2 specifically recognizes p27 in a phosphorylation-dependent manner and targets it for ubiquitination by Skp2-containing SCF [47]. Notably, an over-expression of Skp2 in prostate cancer tissue has been reported to be associated with a low level of p27, higher Gleason score, more advanced pathological stage, higher Ki-67 index, and poor prognosis [57]. Pirh2, a RING-H2-type ubiquitin ligase, also possesses the capability to ubiquitinate p27 for its degradation. Knockdown of Pirh2 compromises p27 degradation and inhibits cell cycle progression at G_1_-S transition [55]. In several malignancies, high levels of Pirh2 are correlated with low p27 expression and associated with a poor prognosis [58–60]. CRM1 is a carrier protein, which transports proteins from nucleus to cytoplasm in a nuclear export signal dependent manner [56]. It can also bind to phosphorylated p27 at Ser^10^ for a nuclear export at G_0_-G_1_ transition, thus lifting its inhibitory effect on CDK2 activity [61]. Following the nuclear export p27 is subsequently subjected to degradation [62]. The CRM1 levels have been reported to be correlated with poor clinical outcome in several malignances [63–67].

In this study, we found that p27 levels were elevated while Skp2 levels diminished in quiescent prostate cancer cells. Under the conditions that the quiescent cancer cells were allowed to re-enter the cell cycle, Efipladib suppressed a recovery of Skp2 from a low levels seen in the quiescent state. The treatment also precluded a diminishment of p27 from high levels and retained it in the nuclei of the cells. It is worthy to note that nuclear retention of p27 due to its mutation from Ser^10^ to Ala^10^ is refractory to Ras-induced lung tumor [39]. Counter-intuitively, CRM1 levels in the cancer cells were largely unchanged in the quiescent status compared to the proliferating state. Similarly, its levels were not affected when the quiescent cancer cells were induced to re-enter the cell cycle either in the presence or absence of Efipladib. Intriguingly, Pirh2 levels were reduced in quiescent LNCaP cells but largely unchanged in quiescent PC-3 cells compared to their proliferative states. During the cell cycle re-entry, inhibition of cPLA_2_α resulted in increased Pirh2 levels in LNCaP cells but reduced levels in PC-3 cells. It is unclear if this phenomenon is related to the difference in p53 status between these two cell lines.

As inhibition of cPLA_2_α with Efipladib led to a persistent blockade of cell cycle re-entry by quiescent prostate cancer cells under *in vitro* conditions, we sought to determine if there was any negative impact of cPLA_2_α inhibition on the anchorage-independent growth of prostate cancer cells under *in vivo* conditions. Since there is no optimal *in vivo* model for determination of cell cycle re-entry, we treated quiescent PC-3 cells expressing GFP with or without Efipladib for 5 days upon cell cycle re-entry *in vitro* and then implanted them subcutaneously to athymic nude mice. One week after cell implantation, the amount of GFP declined by ∼51% in the vehicle control compared to its initial GFP level at day 0. However the amount of GFP dropped more than 84% in the treatment cohort, despite the baseline GFP amount being similar for both cohorts. Thereafter, the diminished number of cells progressively recovered in both cohorts. To our knowledge, this is the first time to show that there is a transient decline in the number of prostate cancer cells following implantation prior to a resurgent growth of implanted cancer cells. From then on, a significant difference of the GFP amount between the cohorts remained over the most part of the monitoring course, which is in congruous with the *in vitro* observation that cPLA_2_α inhibition blocks cell cycle re-entry by quiescent prostate cancers.

In summary, we have demonstrated that pharmacological blockade of cPLA_2_α inhibits the cell cycle re-entry of quiescent prostate cancer cells. The mechanisms underlying the observation are likely due to a perturbation in scheduled turnover of Skp2 required for degradation of p27, and impediment in the re-accumulation of cyclins/CDKs and the components of Pre-RC complex. The significance of cPLA_2_α in the cell cycle re-entry recognized in this study may possess a clinical value in the formulation of a more effective approach to prevent the progression and recurrence of prostate cancer.

## MATERIALS AND METHODS

### Cell culture

Bone metastasized prostate cancer cells (PC-3, CRL-1435, ATCC) and lymph node metastasized prostate cancer cells (LNCaP, CRL-1740, ATCC) were grown in RPMI 1640 supplemented with 10% fetal calf serum (Hyclone), penicillin at 100 units/mL and streptomycin at 100 μg/mL. The cells were cultured at 37°C in an incubator providing a humidified environment in the presence of 5% CO_2_.

### Synchronization of prostate cancer cells into quiescence

To synchronize cells by contact inhibition, PC-3 cells were cultured in T75 flasks and allowed to reach a complete confluence. Thereafter, the confluence was maintained for 3–7 days and the medium was changed every 2–3 days. To synchronize cells by serum withdrawal, LNCaP cells were first cultured in T75 flasks and allowed to reach 70–80% confluence. Thereafter, the serum-containing medium was replaced by serum-free medium for 3–7 days without medium change. To determine the degree of cell quiescence, PC-3 and LNCaP cells were harvested and analyzed for cell cycle phase distribution and Ki-67 expression.

### Treatment with efipladib upon cell cycle re-entry

Efipladib was first dissolved in DMSO at 100 mM and stored at −80°C. To initiate treatment, PC-3 cells with 3 day-confluence were re-plated at low confluence, whereas LNCaP cells with 7 day serum withdrawal were re-plated in the presence of serum. Efipladib at 25 μM for PC-3 cells and 20 μM for LNCaP cells or DMSO as a vehicle control was then added to the medium and the cells were treated for 3 or 5 days. The adhesion of experimental cells was monitored microscopically during the course of treatment. Cells were subsequently collected and assigned for SYBRGreen assay, flow cytometric analysis, immunocytochemistry and immunoblotting.

### SYBRGreen assay

The quiescent prostate cancer cells were trypsinized and plated in 96-well plates at 6,000 cells per well for PC-3 cells and at 10,000 cells per well for LNCaP cells in 150 μL of medium supplemented with serum. To treat cells with Efipladib, 150 μL of the medium containing the drug at double strength relative to each indicated working concentration was added to the wells. The same number of cells was aliquoted as a baseline and stored at −80°C until use. Three days later, the medium was gently aspirated from the plates and 100 μL of lysis buffer containing SYBRGreen (S-7563, Invitrogen) at 1:10,000 v/v dilution was added to each well. The lysis buffer was made up of 9 portions of buffer A (10 mM Tris-pH 7.5 and 2 M NaCl) and 1 portion of buffer B (100 mM Tris-pH 7.5, 200 mM disodium EDTA and 10% Triton X-100). The cells were then lyzed in the dark for 30 min. The frozen aliquots used as baselines were thawed at room temperature, lyzed in the same buffer and transferred to the same treatment plate containing the same type of cells. The fluorescence intensity of SYBRGreen-stained DNA was measured using a plate reader (FLUOstar Omega, BMG Labtech) and the amount of DNA over the study period was determined by subtracting baseline fluorescence intensity from each treatment.

### Flow cytometric analysis

The cell cycle phase distribution was determined by flow cytometric analysis of cellular DNA content. The cells were treated in T25 flasks for the indicated time period. Thereafter, the cells were harvested by trypsinization and fixed by cold 70% v/v ethanol in PBS at 4°C. The fixed cells were washed in PBS and stained in PBS containing 100 μg/mL RNase (R6513, Sigma-Aldrich) and 20 μg /mL propidium iodide (P4170, Sigma-Aldrich) at 37°C for 60 min. The stained cells were washed and re-suspended in PBS. For each sample, at least 20,000 events were acquired to assess cellular DNA content using a flow cytometer (FACSCalibur) equipped with CellQuest Pro software (BD Biosciences) and the acquired data were analyzed using FlowJo software (version 8.1.1).

### BrdU incorporation assay

As an additional approach to monitor DNA synthesis besides SYBRGreen assay, the experimental cells were incubated with 10 μM BrdU (B9285, Sigma Aldrich) for 6 hours prior to collection and then processed for immunocytochemical staining to detect and quantify the cells with incorporated BrdU.

### Immunocytochemistry and quantification

The experimental cells in T75 flasks were harvested, fixed in 10% v/v neutral buffered formalin at 4°C, clotted into 1% w/v/agarose and processed for paraffin blocks. Sections of 5 μm thickness were cut and incubated at 60°C for 1 h, deparaffinized in xylene, re-hydrated in graded ethanol and distilled water, and subjected to antigen retrieval in Tris–EDTA solution using a microwave oven [68]. The sections were blocked with 10% v/v goat serum and then incubated either with primary antibody to Ki-67 (RM-9106-S, Thermo Scientific) or the antibody to phospho-Rb protein at Ser^807/811^ (9308, Cell Signaling Technology) for 20 h at 4°C. After being rinsed in Tris-buffered saline containing 0.05% v/v Tween-20, each section was sequentially labelled with a biotinylated secondary antibody (BA-1000) and Vectastain ABC kit (PK-4000) from Vector Laboratories. Thereafter, the labelled proteins were visualized with 3, 3′-diaminobenzidine tetrahydrochloride (K3468; Dako). The sections were then counterstained with haematoxylin and cover-slipped. For the detection of incorporated BrdU in the cells, the sections were treated with 2 N HCl for 20 min following rehydration and antigen retrieval. The sections were then rinsed with distilled water and incubated with TBST for 10 min prior to blocking with 10% v/v goat serum and subsequent incubation with primary antibody to BrdU (B2531, Sigma-Aldrich) for 20 h at 4°C. After washing with TBST, each section was successively labelled with a biotinylated secondary antibody (BA-9200) and Vectastain ABC kit from Vector Laboratories prior to coloration of immunolabeling with 3, 3′-diaminobenzidine tetrahydrochloride.

The immuno-stained sections were scanned by an automated cellular imaging system (ACIS III; Dako) to acquire digital images. For quantification, two color thresholds were chosen on the images to distinguish the positive (brown) from negative (blue) cells. Ten areas were randomly selected for each treatment to determine the number of positive and negative cells. The percentage of positive cells of each treatment was calculated using following formula: positive cells/ (positive cells + negative cells) × 100%.

### Immunofluorescence and quantification

The experimental cells in T75 flasks were collected, fixed in 10% neutral buffered formalin at 4°C, clotted into 1% w/v agarose and processed for paraffin blocks. Sections with 3 μm thicknesses were cut and incubated at 60°C for 1 h, deparaffinized in xylene, re-hydrated in graded ethanol and distilled water, and subjected to antigen retrieval in Tris–EDTA solution using a microwave oven. The sections were blocked with 10% v/v goat serum and then incubated with primary antibody to p27 (sc-528, Santa Cruz Biotechnology) for 20 h at 4°C. After being rinsed in TBST, each section was subsequently labelled with a secondary antibody conjugated with Alaxa488 (A-21441, Invitrogen). The labeled sections were then counterstained for nuclei with 4′, 6-Diamidino-2-phenylindole dihydrochloride (D8417, Sigma-Aldrich) and cover-slipped.

The images of both p27 labelling and nuclear staining of each sample were acquired from the labeled sections using an Olympus microscope (AX70 Provis) equipped with Olympus DP controller and DP manager. The acquired images of p27 labeling and nuclear staining were superimposed and saved as JPEG files together with each image of single color. The saved files were then analyzed using ImageJ 1.46 (National Institute of Health). A threshold was chosen on the images of p27 labeling to distinguish bright signal from dim signals, enabling calculation of the percentage of cells with nuclear p27 positive cells using following formula: (number of nuclei with bright p27/number of nuclei) × 100%. At least 6,000 cells were analyzed for every experimental sample.

### Immunoblotting

The cells were treated in 6-well plates with Efipladib at 25 μM for PC-3 cells and 20 μM for LNCaP cells or DMSO as a vehicle control for 3 or 5 days. Thereafter, the cell lysates were prepared with a lysis buffer (50 mM Tris-pH 8, 150 mM NaCl, 1% v/v Igepal CA-630, 0.5% w/v sodium deoxycholate, 0.1% w/v sodium dodecyl sulfate) supplemented with a protease inhibitor cocktail (11836145001, Roche Supplied Science). Protein concentration was quantified by Bio-Rad DC Protein Assay. Proteins in the lysates were separated by SDS-PAGE and then transferred onto a nitrocellulose membrane (RPN303E, Amersham Biosciences). To perform immunolabeling, the membranes were blocked with 1% w/v non-fat milk in phosphate buffered saline with 0.5% v/v Tween-20 (PBST) for 30 minutes and then incubated with primary antibodies for 1–2 days at 4°C. The blots were washed in PBST, and incubated with an appropriate secondary antibody conjugated with peroxidase (Sigma-Aldrich) at room temperature for 3 h. The membranes were rinsed in PBST and incubated with SuperSignal West Pico Chemiluminescent Substrate (34078, Thermo Scientific). The luminescence emitted from labeled proteins was recorded using a gel documentation and analysis system equipped with a CCD camera (Syngene). The primary antibodies against p27 (sc-528), CRM1 (sc-74454), Pirh2 (sc-67033), Skp2 (sc-7164), Cyclin E (sc-198), CDK2 (sc-748), CDK4 (sc-749), CDC6 (sc-9964), MCM7 (sc-22782), ORC6 (sc-20636), PCNA (sc-56), cPLA_2_α (sc-454) and phospho-cPLA_2_ at Ser^505^ (sc-34391-R) were purchased from Santa Cruz Biotechnology. The antibody to cyclin D1 (C7464) was purchased from Sigma-Aldrich, and the antibody to α-tubulin (ab7291) from Abcam.

Immunoblot images were exported in the format of tagged image file and quantified using ImageJ 1.46 software (National Institute of Health). Except for phospho-cPLA_2_α, the immunblot bands were normalized to α-tubulin as a loading control. To analyze the levels of phospho-cPLA_2_α, phospho-cPLA_2_α was first normalized to total cPLA_2_α and then to α-tubulin. The baseline values of non-synchronized condition were set to 100% and those obtained from other conditions were presented as the percentage of the baseline values.

### Monitoring of xenograft derived from implanted cancer cells

PC-3 cells were stably transduced with the GFP-P2A-luc lentiviral vector [69]. The cells were selected by FACS based on high GFP expression (≥10^3^ relative fluorescence units). Prior to the *in vivo* study, GFP expression was analyzed again by flow cytometry to confirm maintenance of high GFP expression in propagated cells. Consistently, about 93% of the cells are positive for GFP with parental PC-3 cells as a negative control, and approximately 90% of these positive cells displayed high GFP expression (≥10^3^ relative fluorescence units). The GFP-expressing PC-3 cells were rendered quiescent by contact-inhibition before being re-plated at a low density (1:6 split) in the presence of either DMSO or 25 μM Efipladib for 5 days, prior to implantation into 8 week-old male nude mice. All *in vivo* experiments were conducted with approval from the Animal Ethics Committee at The University of Sydney. The cells were collected by trysinization, re-suspended in the culturing medium at 5 × 10^6^ cells/mL and injected into mice at 1 × 10^6^ cells per flank. To measure GFP intensity of injected sites, mice were anesthetized with isoflurane (Baxter Healthcare) and 2% v/v oxygen delivered by a Komesaroff anesthetic machine (Medical Developments Australia). The anesthesia was first induced with 5% v/v isoflurane and then maintained with 2% v/v isoflurane in the presence of 2% v/v oxygen throughout the imaging process. The images of GFP were acquired using an *in vivo* imaging system (*In-Vivo* MS FX Pro, Bruker) equipped with imaging software (Bruker MI SE). Thereafter, the mice were allowed to recover from anesthesia. The acquired images were exported in a green-on-black format (color range from 200 to 20000 with gamma setting 1.2) and saved as JPEG files. The recorded GFP was then quantified using ImageJ 1.46 software. To this end, the GFP spot in each image was first traced along its border and both its size and average fluorescence intensity were measured. Then, the trace was moved to an area adjacent to GFP spot and background noise was also measured. The genuine GFP amount in each implanted site was calculated as follows: (the fluorescence intensity of GFP spot – the fluorescence intensity of background) × the size of GFP spot.

### Statistical analysis

The statistical software NCSS version 12.0 was employed for statistical analysis. One-Way ANOVA was used to determine difference among groups. Fisher's LSD Multiple-Comparison Test was used for pairwise comparisons (*p* < 0.05 was considered significant). Data that failed the test for normal distribution or homogeneous variance were analyzed with the Kruskal-Wallis One-Way ANOVA on Ranks followed by Kruskal-Wallis Multiple-Comparison Z-Value Test (z-value of >1.96 was considered significant).

## SUPPLEMENTARY FIGURES



## Abbreviations

CDK: cyclin-dependent kinase
pRb: retinoblastoma protein
Skp2: S-phase kinase-associated protein 2
ORC: origin replication complex
CDC6: cell division cycle 6
MCM: minichromosome maintenance
PCNA: proliferating cell nuclear antigen
CRM1: Chromosomal region maintenance 1
Pirh2: P53-induced RING-H2 protein

## References

[1] Brawley O (2012). Prostate cancer epidemiology in the United States. World J Urol.

[2] Center MM, Jemal A, Lortet-Tieulent J, Ward E, Ferlay J, Brawley O, Bray F (2012). International Variation in Prostate Cancer Incidence and Mortality Rates. Eur Urol.

[3] Pound CR, Partin AW, Eisenberger MA, Chan DW, Pearson JD, Walsh PC (1999). Natural history of progression after PSA elevation following radical prostatectomy. JAMA.

[4] Freedland SJ, Humphreys EB, Mangold LA, Eisenberger M, Dorey FJ, Walsh PC, Partin AW (2005). Risk of prostate cancer-specific mortality following biochemical recurrence after radical prostatectomy. JAMA.

[5] Trock BJ, Han M, Freedland SJ, Humphreys EB, DeWeese TL, Partin AW, W PC (2008). Prostate cancer-specific survival following salvage radiotherapy vs observation in men with biochemical recurrence after radical prostatectomy. JAMA.

[6] Jackson RC (1989). The problem of the quiescent cancer cell. Adv Enzyme Regul.

[7] Desoize B, Jardillier J (2000). Multicellular resistance: a paradigm for clinical resistance?. Crit Rev Oncol Hematol.

[8] Berges RR, Vukanovic J, Epstein JI, CarMichel M, Cisek L, Johnson DE, Veltri RW, Walsh PC, Isaacs JT (1995). Implication of cell kinetic changes during the progression of human prostatic cancer. Clin Cancer Res.

[9] Keshari KR, Tsachres H, Iman R, Delos Santos L, Tabatabai ZL, Shinohara K, Vigneron DB, Kurhanewicz J (2011). Correlation of phospholipid metabolites with prostate cancer pathologic grade, proliferative status and surgical stage - impact of tissue environment. NMR Biomed.

[10] Khatami A, Hugosson J, Wang W, Damber JE (2009). Ki-67 in screen-detected, low-grade, low-stage prostate cancer, relation to prostate specific antigen doubling time, Gleason score and prostate-specific antigen relapse after radical prostatectomy. Scand J Urol Nephrol.

[11] Kaiser E CP, Zaky K (1990). Phospholipases in biology and medicine. Clin Biochem.

[12] Dennis EA (1997). The growing phospholipase A2 superfamily of signal transduction enzymes. Trends Biochem Sci.

[13] Tucker DE, Ghosh M, Ghomashchi F, Loper R, Suram S, John BS, Girotti M, Bollinger JG, Gelb MH, Leslie CC (2009). Role of phosphorylation and basic residues in the catalytic domain of cytosolic phospholipase A2alpha in regulating interfacial kinetics and binding and cellular function. J Biol Chem.

[14] McEntee MF, Ziegler C, Reel D, Tomer K, Shoieb A, Ray M, Li X, Neilsen N, Lih FB, O'Rourke D, Whelan J (2008). Dietary n-3 polyunsaturated fatty acids enhance hormone ablation therapy in androgen-dependent prostate cancer. Am J Pathol.

[15] Kelavkar UP, Hutzley J, Dhir R, Kim P, Allen KG, McHugh K (2006). Prostate tumor growth and recurrence can be modulated by the omega-6:omega-3 ratio in diet: athymic mouse xenograft model simulating radical prostatectomy. Neoplasia.

[16] Ghosh J, Myers CE (1997). Arachidonic acid stimulates prostate cancer cell growth: critical role of 5-lipoxygenase. Biochem Biophys Res Commun.

[17] Chaudry AA, Wahle KW, McClinton S, Moffat LE (1994). Arachidonic acid metabolism in benign and malignant prostatic tissue *in vitro*: effects of fatty acids and cyclooxygenase inhibitors. Int J Cancer.

[18] Pruthi RS, Derksen JE, Moore D, Carson CC, Grigson G, Watkins C, Wallen E (2006). Phase II trial of celecoxib in prostate-specific antigen recurrent prostate cancer after definitive radiation therapy or radical prostatectomy. Clin Cancer Res.

[19] Smith MR, Manola J, Kaufman DS, Oh WK, Bubley GJ, Kantoff PW (2006). Celecoxib versus placebo for men with prostate cancer and a rising serum prostate-specific antigen after radical prostatectomy and/or radiation therapy. J Clin Oncol.

[20] Xu B GL, Wang L, Tang G, He M, Yu Y, Ni X, Sun Y (2013). Effects of platelet-activating factor and its differential regulation by androgens and steroid hormones in prostate cancers. Br J Cancer.

[21] Jan CR CY (2004). Novel effect of Y-24180, a presumed specific platelet activation factor receptor antagonist, on Ca2+ levels and growth of human prostate cancer cells. Cell Signal.

[22] Kramer RM, Sharp JD (1997). Structure, function and regulation of Ca2+-sensitive cytosolic phospholipase A2 (cPLA2). FEBS Lett.

[23] McKew JC, Lee KL, Shen MW, Thakker P, Foley MA, Behnke ML, Hu B, Sum FW, Tam S, Hu Y, Chen L, Kirincich SJ, Michalak R, Thomason J, Ipek M, Wu K (2008). Indole cytosolic phospholipase A2 alpha inhibitors: discovery and *in vitro* and *in vivo* characterization of 4-{3-[5-chloro-2-(2-{[(3,4-dichlorobenzyl)sulfonyl]amino}ethyl)-1-(diphenylmethyl)-1H-indol-3-yl]propyl benzoic acid, efipladib. J Med Chem.

[24] Gerdes J, Lemke H, Baisch H, Wacker HH, Schwab U, Stein H (1984). Cell cycle analysis of a cell proliferation-associated human nuclear antigen defined by the monoclonal antibody Ki-67. J Immunol.

[25] Duchrow M, Schlüter C, Key G, Kubbutat MH, Wohlenberg C, Flad HD, J JG (1995). Cell proliferation-associated nuclear antigen defined by antibody Ki-67: a new kind of cell cycle-maintaining proteins. Arch Immunol Ther Exp (Warsz).

[26] Stoeber K, Mills AD, Kubota Y, Krude T, Romanowski P, Marheineke K, Laskey RA, Williams GH (1998). Cdc6 protein causes premature entry into S phase in a mammalian cell-free system. EMBO J.

[27] Lavoie JN, L'Allemain G, Brunet A, Müller R, Pouysségur J (1996). Cyclin D1 expression is regulated positively by the p42/p44MAPK and negatively by the p38/HOGMAPK pathway. J Biol Chem.

[28] Harbour JW, Luo RX, Dei Santi A, Postigo AA, Dean DC (1999). Cdk phosphorylation triggers sequential intramolecular interactions that progressively block Rb functions as cells move through G1. Cell.

[29] DeGregori J, Kowalik T, Nevins JR (1995). Cellular targets for activation by the E2F1 transcription factor include DNA synthesis- and G1/S-regulatory genes. Mol Cell Biol.

[30] Johnson DG, Schwarz JK, Cress WD, Nevins JR (1993). Expression of transcription factor E2F1 induces quiescent cells to enter S phase. Nature.

[31] Coller HA (2007). What's taking so long? S-phase entry from quiescence versus proliferation. Nat Rev Mol Cell Biol.

[32] Kingsbury SR, Loddo M, Fanshawe T, Obermann EC, Prevost AT, Stoeber K, Williams GH (2005). Repression of DNA replication licensing in quiescence is independent of geminin and may define the cell cycle state of progenitor cells. Exp Cell Res.

[33] Prelich G TC, Kostura M, Mathews MB, So AG, Downey KM, Stillman B (1987). Functional identity of proliferating cell nuclear antigen and a DNA polymerase-delta auxiliary protein. Nature.

[34] Rivard N, L'Allemain G, Bartek J, Pouysségur J (1996). Abrogation of p27Kip1 by cDNA antisense suppresses quiescence (G0 state) in fibroblasts. J Biol Chem.

[35] Nakanishi M, Rosenberg DW (2006). Roles of cPLA2alpha and arachidonic acid in cancer. Biochimica et biophysica acta.

[36] Hua S, Yao M, Vignarajan S, Witting P, Hejazi L, Gong Z, Teng Y, Niknami M, Assinder S, Richardson D, Dong Q (2013). Cytosolic phospholipase A2alpha sustains pAKT, pERK and AR levels in PTEN-null/mutated prostate cancer cells. Biochimica et biophysica acta.

[37] Zheng Z, He X, Xie C, Hua S, Li J, Wang T, Yao M, Vignarajan S, Teng Y, Hejazi L, Liu B, Dong Q (2014). Targeting cytosolic phospholipase A2 alpha in colorectal cancer cells inhibits constitutively activated protein kinase B (AKT) and cell proliferation. Oncotarget.

[38] Patel MI, Singh J, Niknami M, Kurek C, Yao M, Lu S, Maclean F, King NJ, Gelb MH, Scott KF, Russell PJ, Boulas J, Dong Q (2008). Cytosolic phospholipase A2-alpha: a potential therapeutic target for prostate cancer. Clin Cancer Res.

[39] Besson A, Gurian-West M, Chen XY, Kelly-Spratt KS, Kemp CJ, Roberts JM (2006). A pathway in quiescent cells that controls p27(Kip1) stability, subcellular localization, and tumor suppression. Gene Dev.

[40] Medina R, Zaidi SK, Liu CG, Stein JL, van Wijnen AJ, Croce CM, Stein GS (2008). MicroRNAs 221 and 222 bypass quiescence and compromise cell survival. Cancer research.

[41] Ladha MH, Lee KY, Upton TM, Reed MF, Ewen ME (1998). Regulation of exit from quiescence by p27 and cyclin D1-CDK4. Mol Cell Biol.

[42] Drobnjak M, Osman I, Scher HI, Fazzari M, Cordon-Cardo C (2000). Overexpression of cyclin D1 is associated with metastatic prostate cancer to bone. Clin Cancer Res.

[43] Zhang T, Nanney LB, Luongo C, Lamps L, Heppner KJ, DuBois RN, Beauchamp RD (1997). Concurrent overexpression of cyclin D1 and cyclin-dependent kinase 4 (Cdk4) in intestinal adenomas from multiple intestinal neoplasia (Min) mice and human familial adenomatous polyposis patients. Cancer research.

[44] Keyomarsi K, Tucker SL, Buchholz TA, Callister M, Ding Y, Hortobagyi GN, Bedrosian I, Knickerbocker C, Toyofuku W, Lowe M, Herliczek TW, Bacus SS (2002). Cyclin E and survival in patients with breast cancer. N Engl J Med.

[45] Liang Y, Gao H, Lin SY, Goss JA, Brunicardi FC, Li K (2010). siRNA-based targeting of cyclin E overexpression inhibits breast cancer cell growth and suppresses tumor development in breast cancer mouse model. PLoS One.

[46] Sheaff RJ, Groudine M, Gordon M, Roberts JM, Clurman BE (1997). Cyclin E-CDK2 is a regulator of p27Kip1. Genes Dev.

[47] Carrano AC, Eytan E, Hershko A, Pagano M (1999). SKP2 is required for ubiquitin-mediated degradation of the CDK inhibitor p27. Nat Cell Biol.

[48] Geng Y, Yu Q, Sicinska E, Das M, Schneider JE, Bhattacharya S, Rideout WM, Bronson RT, Gardner H, Sicinski P (2003). Cyclin E ablation in the mouse. Cell.

[49] Geng Y, Lee YM, Welcker M, Swanger J, Zagozdzon A, Winer JD, Roberts JM, Kaldis P CB, Sicinski P (2007). Kinase-independent function of cyclin E. Mol Cell.

[50] Semple JW, Duncker BP (2004). ORC-associated replication factors as biomarkers for cancer. Biotechnol Adv.

[51] Wang X, Hickey RJ, Malkas LH, Koch MO, Li L, Zhang S, Sandusky GE, Grignon DJ, Eble JN, Cheng L (2011). Elevated expression of cancer-associated proliferating cell nuclear antigen in high-grade prostatic intraepithelial neoplasia and prostate cancer. The Prostate.

[52] Hengst L, Reed SI (1996). Translational control of p27Kip1 accumulation during the cell cycle. Science.

[53] Coats S, Flanagan WM, Nourse J, Roberts JM (1996). Requirement of p27Kip1 for restriction point control of the fibroblast cell cycle. Science.

[54] Sgambato A, Cittadini A, Faraglia B, Weinstein IB (2000). Multiple functions of p27(Kip1) and its alterations in tumor cells: a review. J Cell Physiol.

[55] Hattori T, Isobe T, Abe K, Kikuchi H, Kitagawa K, Oda T, Uchida C, Kitagawa M (2007). Pirh2 promotes ubiquitin- dependent degradation of the cyclin-dependent kinase inhibitor p27Kip1. Cancer research.

[56] Fukuda M, Asano S, Nakamura T, Adachi M, Yoshida M, Yanagida M, Nishida E (1997). CRM1 is responsible for intracellular transport mediated by the nuclear export signal. Nature.

[57] Nguyen PL, Lin DI, Lei J, Fiorentino M, Mueller E, Weinstein MH, Pagano M, Loda M (2011). The impact of Skp2 overexpression on recurrence-free survival following radical prostatectomy. Urol Oncol.

[58] Shimada M, Kitagawa K, Dobashi Y, Isobe T, Hattori T, Uchida C, Abe K, Kotake Y, Oda T, Suzuki H, Hashimoto K, Kitagawa M (2009). High expression of Pirh2, an E3 ligase for p27, is associated with low expression of p27 and poor prognosis in head and neck cancers. Cancer science.

[59] Huang X, Qian X, Cheng C, He S, Sun L, Ke Q, Zhang L, Pan X, He F, Wang Q, Meng J, Ni R, Shen A (2011). Expression of Pirh2, a p27(Kip1) ubiquitin ligase, in hepatocellular carcinoma: correlation with p27(Kip1) and cell proliferation. Human pathology.

[60] Wu XR, Sha JJ, Liu DM, Chen YH, Yang GL, Zhang J, Chen YY, Bo JJ, Huang YR (2013). High expression of P53-induced Ring-h2 protein is associated with poor prognosis in clear cell renal cell carcinoma. European journal of surgical oncology: the journal of the European Society of Surgical Oncology and the British Association of Surgical Oncology.

[61] Connor MK, Kotchetkov R, Cariou S, Resch A, Lupetti R, Beniston RG, Melchior F, Hengst L, Slingerland JM (2003). CRM1/Ran-mediated nuclear export of p27(Kip1) involves a nuclear export signal and links p27 export and proteolysis. Molecular biology of the cell.

[62] Ishida N, Hara T, Kamura T, Yoshida M, Nakayama K, Nakayama KI (2002). Phosphorylation of p27Kip1 on serine 10 is required for its binding to CRM1 and nuclear export. J Biol Chem.

[63] Noske A, Weichert W, Niesporek S, Roske A, Buckendahl AC, Koch I, Sehouli J, Dietel M, Denkert C (2008). Expression of the nuclear export protein chromosomal region maintenance/exportin 1/Xpo1 is a prognostic factor in human ovarian cancer. Cancer.

[64] Yao Y, Dong Y, Lin F, Zhao H, Shen Z, Chen P, Sun YJ, Tang LN, Zheng SE (2009). The expression of CRM1 is associated with prognosis in human osteosarcoma. Oncol Rep.

[65] Shen AG, Wang YC, Zhao YM, Zou L, Sun LL, Cheng C (2009). Expression of Crm1 in Human Gliomas and Its Significance in P27 Expression and Clinical Prognosis. Neurosurgery.

[66] Huang WY, Yue L, Qiu WS, Wang LW, Zhou XH, Sun YJ (2009). Prognostic value of CRM1 in pancreas cancer. Clin Invest Med.

[67] Zhou F, Qiu WS, Yao RY, Xiang JY, Sun XX, Liu SH, Lv J, Yue L (2013). CRM1 is a novel independent prognostic factor for the poor prognosis of gastric carcinomas. Med Oncol.

[68] Pileri SA, Roncador G, Ceccarelli C, Piccioli M, Briskomatis A, Sabattini E, Ascani S, Santini D, Piccaluga PP, Leone O, Damiani S, Ercolessi C, Sandri F, Pieri F, Leoncini L, Falini B (1997). Antigen retrieval techniques in immunohistochemistry: comparison of different methods. J Pathol.

[69] Tiffen JC, Bailey CG, Ng C, Rasko JE, Holst J (2010). Luciferase expression and bioluminescence does not affect tumor cell growth *in vitro* or *in vivo*. Molecular cancer.

