# Supplementary material for: Targeting of cytosolic phospholipase A2α impedes cell cycle re-entry of quiescent prostate cancer cells
Source: Oncotarget. 2015 Sep 24;6(33):34458–74. doi: 10.18632/oncotarget.5277 (PMC4741466; doi:10.18632/oncotarget.5277)
Supplement: Supplementary file 1 [file oncotarget-06-34458-s001.pdf]

SUPPLEMENTARY FIGURES

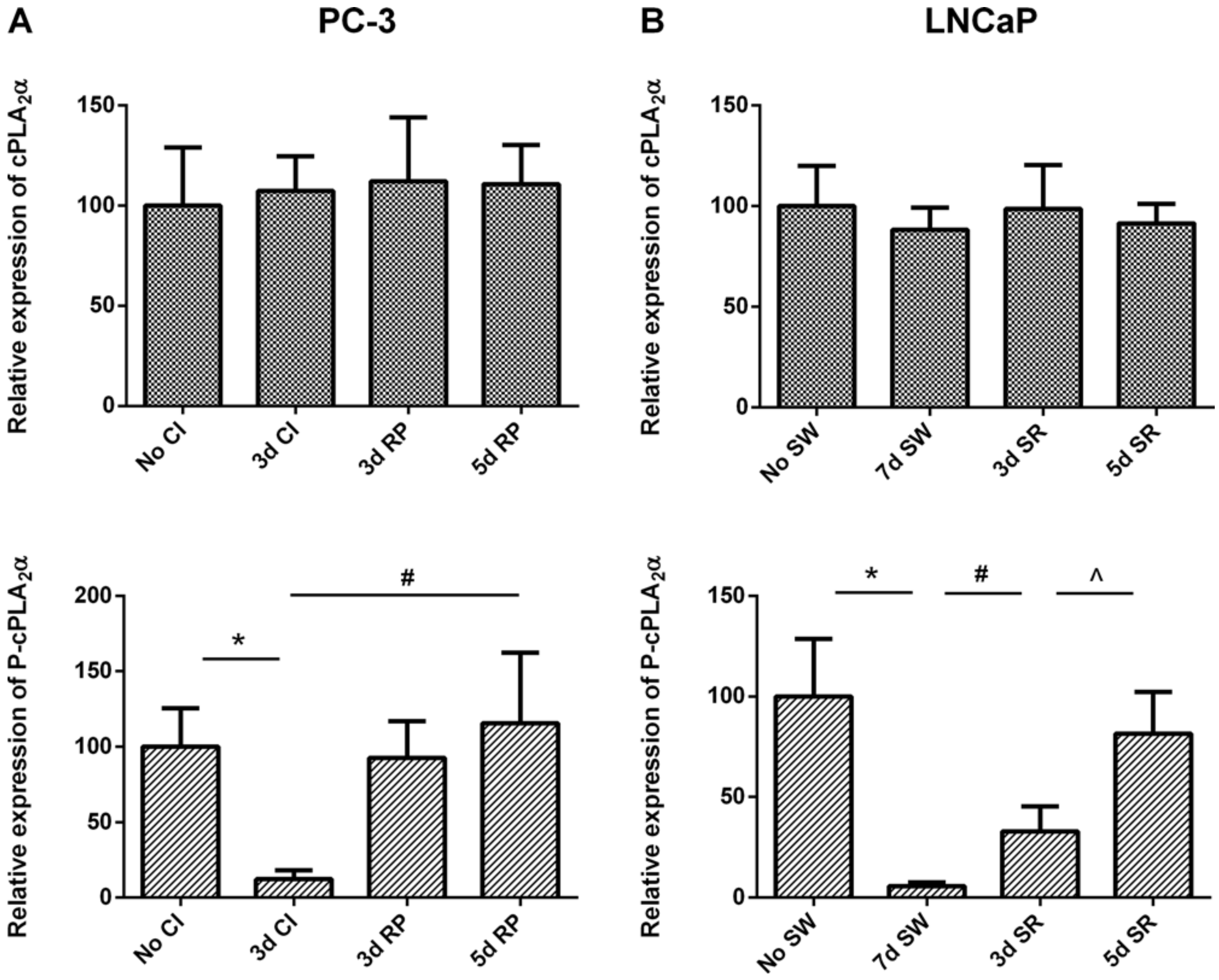

Supplementary Figure S1: Quantification of total and phospho-cPLA$_2$α detected by immunoblotting. The protein of interest was quantified as described in Materials and Methods. The relative expression levels of total and phospho-cPLA$_2$α in both PC-3 and LNCaP cells are expressed as mean ± SD. In **panel A** * Difference ($p < 0.05$) between non-synchronized and proliferating state (no contact inhibition, No CI) and quiescent state (3 day contact inhibition, 3d CI); # Difference ($p < 0.05$) between quiescent state (3d CI) and 3 or 5 days after induction of cell cycle re-entry by replating at low density (3d RP and 5d RP); In **panel B** * Difference ($p < 0.05$) between non-synchronized and proliferating state (no serum withdrawal, No SW) and quiescent state (7 day serum withdrawal, 7d SW); # Difference ($p < 0.05$) between quiescent state (7d SW) and 3 days following induction of cell cycle re-entry by serum restoration (3d SR); ^ Difference ($p < 0.05$) between 3 days and 5 days (5d SR) after induction of cell cycle re-entry. In both panels A and B, $n = 3$ independent experiments.

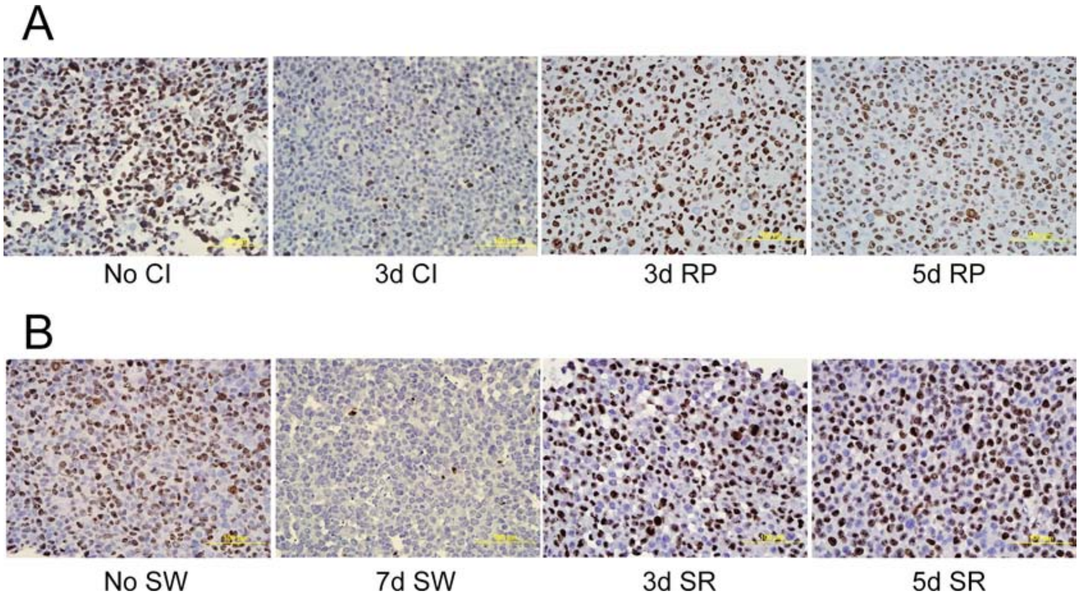

Supplementary Figure S2: Monitoring of cell cycle status by measuring Ki-67 positivity. A. PC-3 cells were rendered to quiescent status by 3 day contact inhibition and then induced to re-enter the cell cycle by re-plating them at a low density (1:6 dilutions) to T75 flasks. **B.** LNCaP cells were made quiescent status by 7 day serum withdrawal and then induced to re-enter the cell cycle by re-plating them in the presence of serum in to T75 flasks. The cells in both A and B were then harvested at the indicated time intervals and processed for immunocytochemical staining of Ki-67. No CI: no contact inhibition. 3d CI: 3 day contact inhibition. 3d RP: 3 days after induction of cell cycle re-entry by replating at low density. 5d RP: 5 days after induction of cell cycle re-entry by replating at low density. No SW: no serum withdrawal. 7d SW: 7 day serum withdrawal. 3d SR: 3 days following induction of cell cycle re-entry by serum restoration. 5d SR: 5 days after induction of cell cycle re-entry by serum restoration.

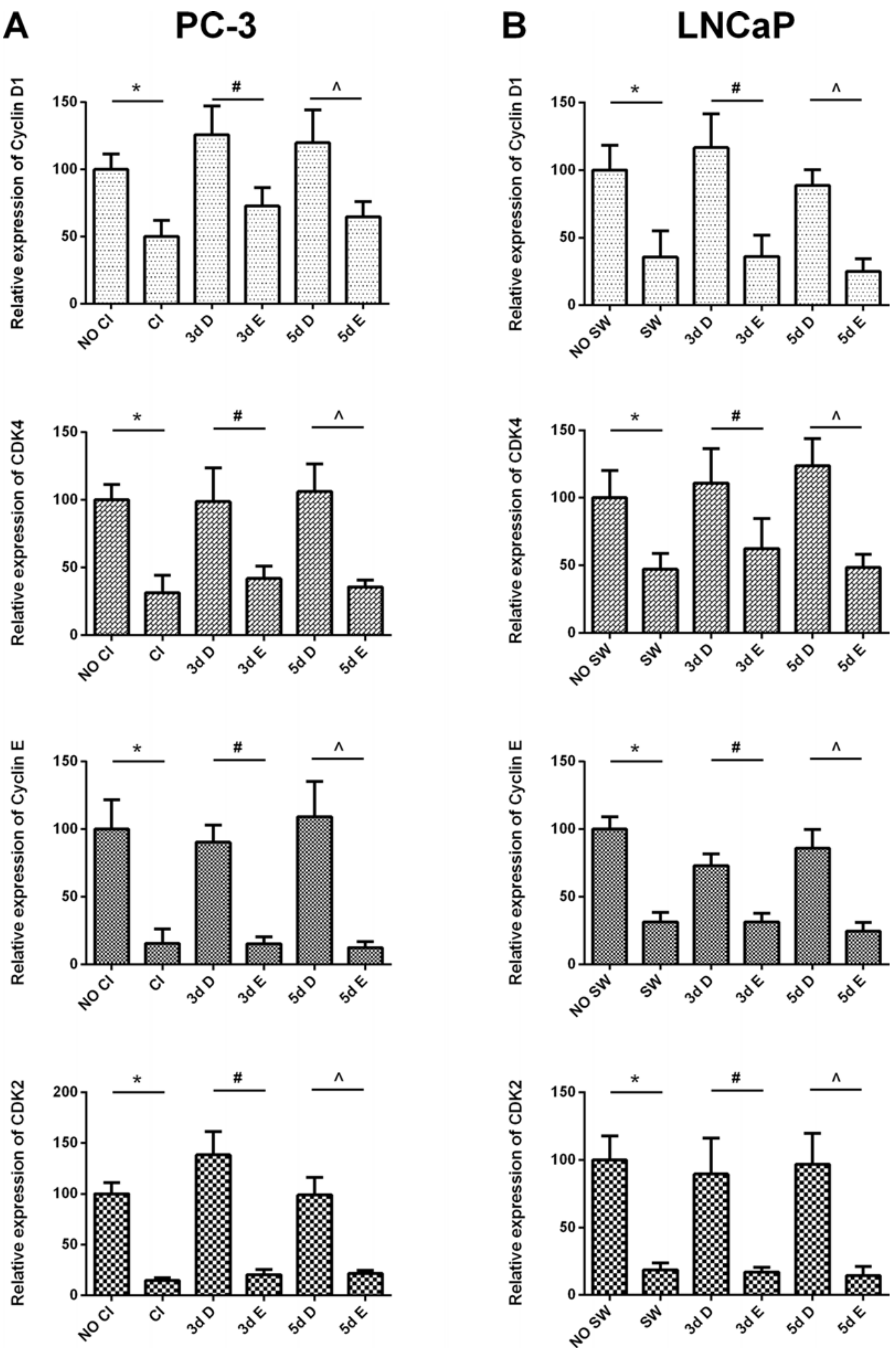

Supplementary Figure S3: Quantification of cyclins and CDKs detected by immunoblotting. The relative expression levels of interested proteins in both PC-3 and LNCaP cells are expressed as mean ± SD. In both **panels A** and **B** * Difference ($p < 0.05$) between non-synchronized and proliferating state (no contact inhibition, No CI or no serum withdrawal, No SW) and quiescent state (contact inhibition, CI or serum withdrawal, SW); # Difference ($p < 0.05$) between vehicle control (DMSO, D) and Efipladib (E) treatment 3 days after induction of cell cycle re-entry; ^ Difference ($p < 0.05$) between vehicle control (DMSO, D) and Efipladib (E) treatment 5 days after induction of cell cycle re-entry; $n = 3$ independent experiments.

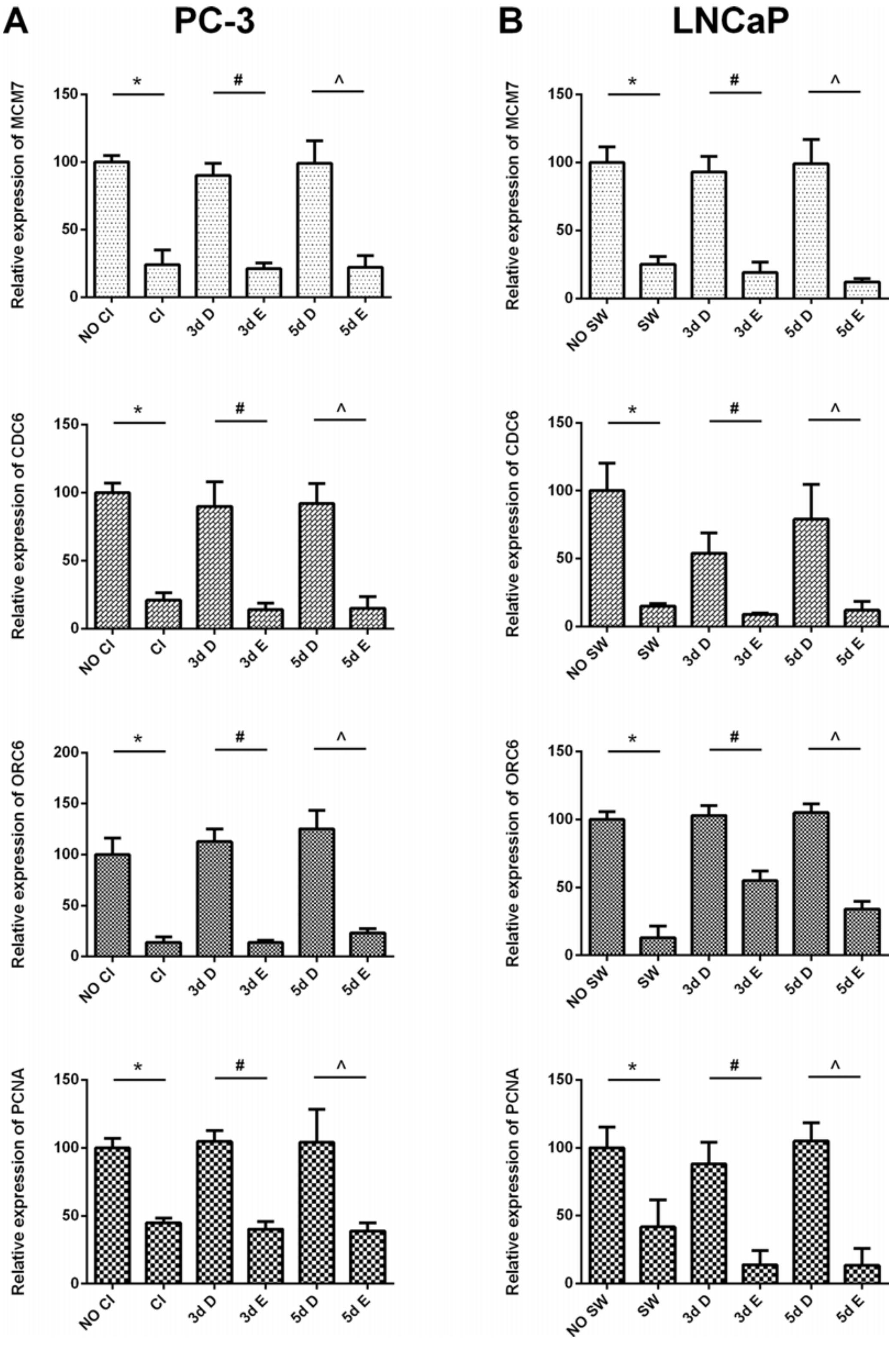

Supplementary Figure S4: Quantification of Pre-RC proteins and PCNA detected by immunoblotting. The relative expression levels of interested proteins in both PC-3 and LNCaP cells are expressed as mean ± SD. In both **panels A** and **B** * Difference ($p < 0.05$) between non-synchronized and proliferating state (no contact inhibition, No CI; or no serum withdrawal, NoSW) and quiescent state (contact inhibition, CI or serum withdrawal, SW); # Difference ($p < 0.05$) between vehicle control (DMSO, D) and Efipladib (E) treatment 3 days after induction of cell cycle re-entry; ^ Difference ($p < 0.05$) between vehicle control (DMSO, D) and Efipladib (E) treatment 5 days after induction of cell cycle re-entry; $n = 3$ independent experiments.

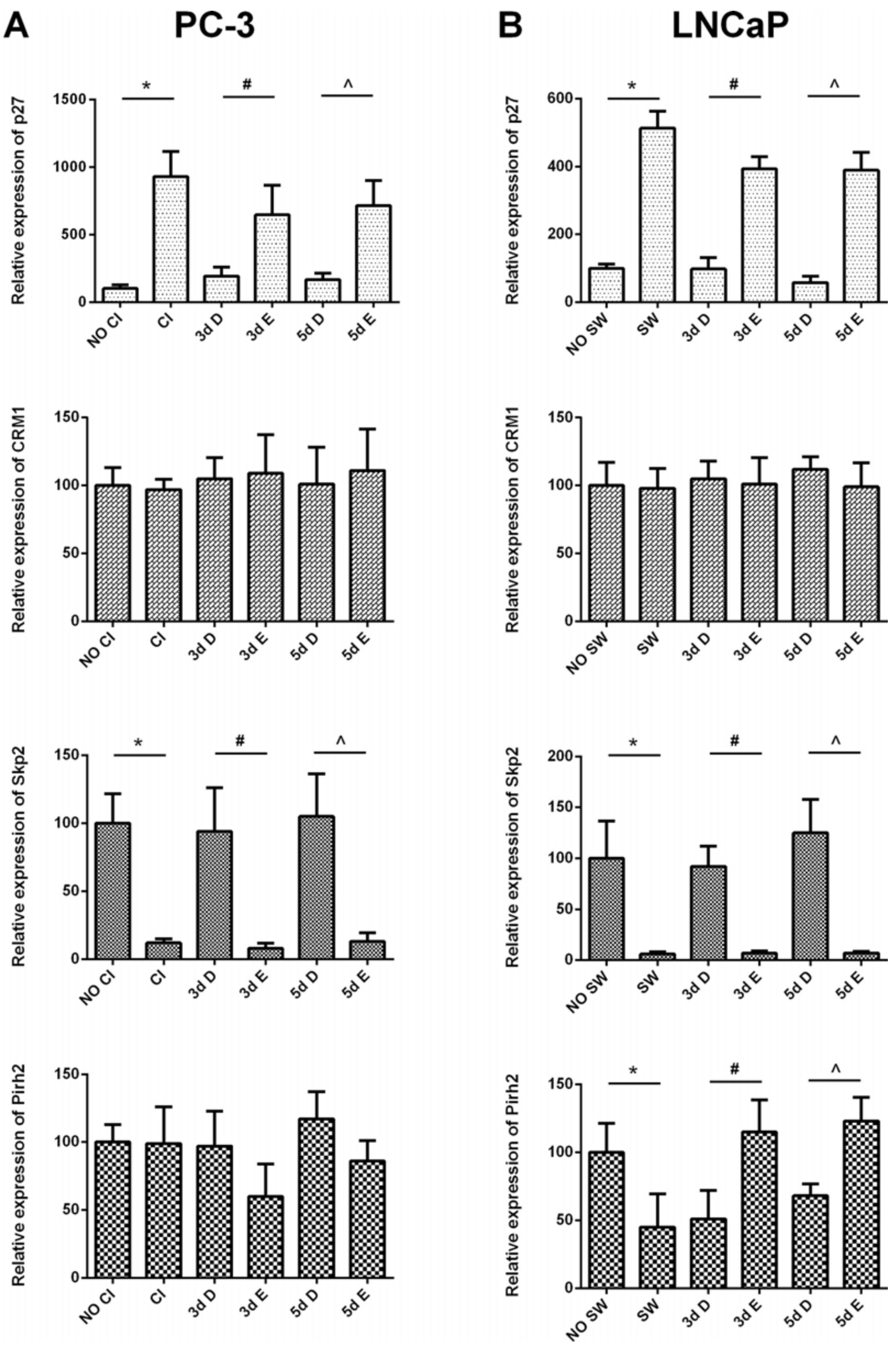

Supplementary Figure S5: Quantification of p27 and its regulators detected by immunoblotting. The relative expression levels of interested proteins in both PC-3 and LNCaP cells are expressed as mean ± SD. In both **panels A** and **B** * Difference ($p < 0.05$) between non-synchronized and proliferating state (no contact inhibition, No CI or no serum withdrawal, NoSW) and quiescent state (contact inhibition, CI or serum withdrawal, SW); # Difference ($p < 0.05$) between vehicle control (DMSO, D) and Efipladib (E) treatment 3 days after induction of cell cycle re-entry; ^ Difference ($p < 0.05$) between vehicle control (DMSO, D) and Efipladib (E) treatment 5 days after induction of cell cycle re-entry; $n = 3$ independent experiments.

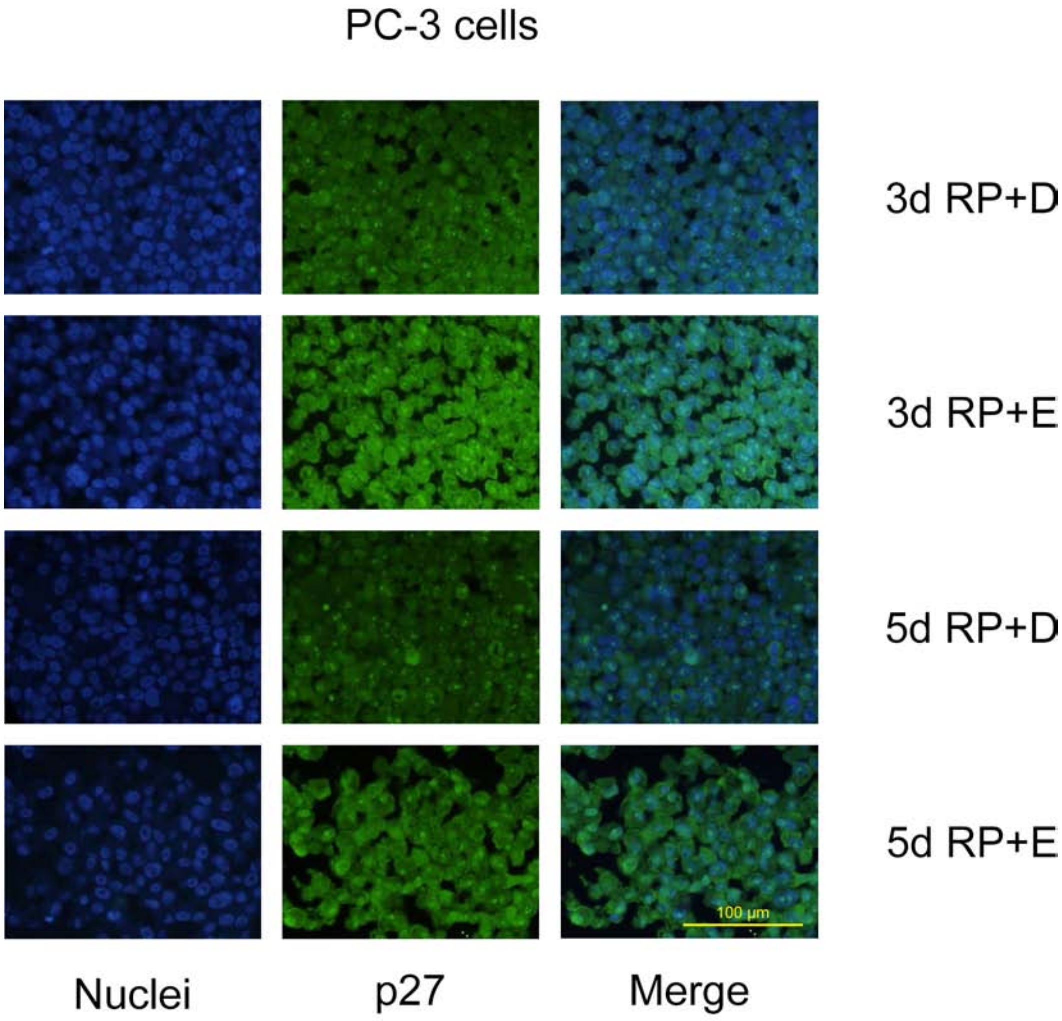

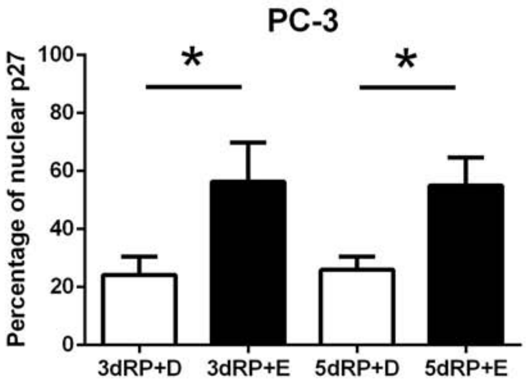

Supplementary Figure S6: Retention of nuclear p27 in PC-3 cells by Efipladib during cell cycle re-entry. Quiescent PC-3 cells were rendered to re-enter cell cycle by re-plating them at a low density in T75 flasks. Either 25 µM Efipladib or DMSO was added upon the induction of cell cycle re-entry. Three days or five days after treatment, the cells were collected for immunofluorescence analysis of p27. The nuclear p27 is illustrated by a superimposition of p27 (green) on the nuclei (blue). The histograms show the percentage of nuclear positive cells and the data represent mean ± SD from one of three experiments. * Different to the vehicle control at each corresponding time interval ($p < 0.01$). 3dRP+D: treatment of re-plated (RP) cells with DMSO (D) for 3 days. 3dRP+E: treatment of re-plated cells with Efipladib (E) for 3 days. 5dRP+D: treatment of re-plated cells with DMSO for 5 days. 5dRP+E: treatment of re-plated cells with Efipladib for 5 days.

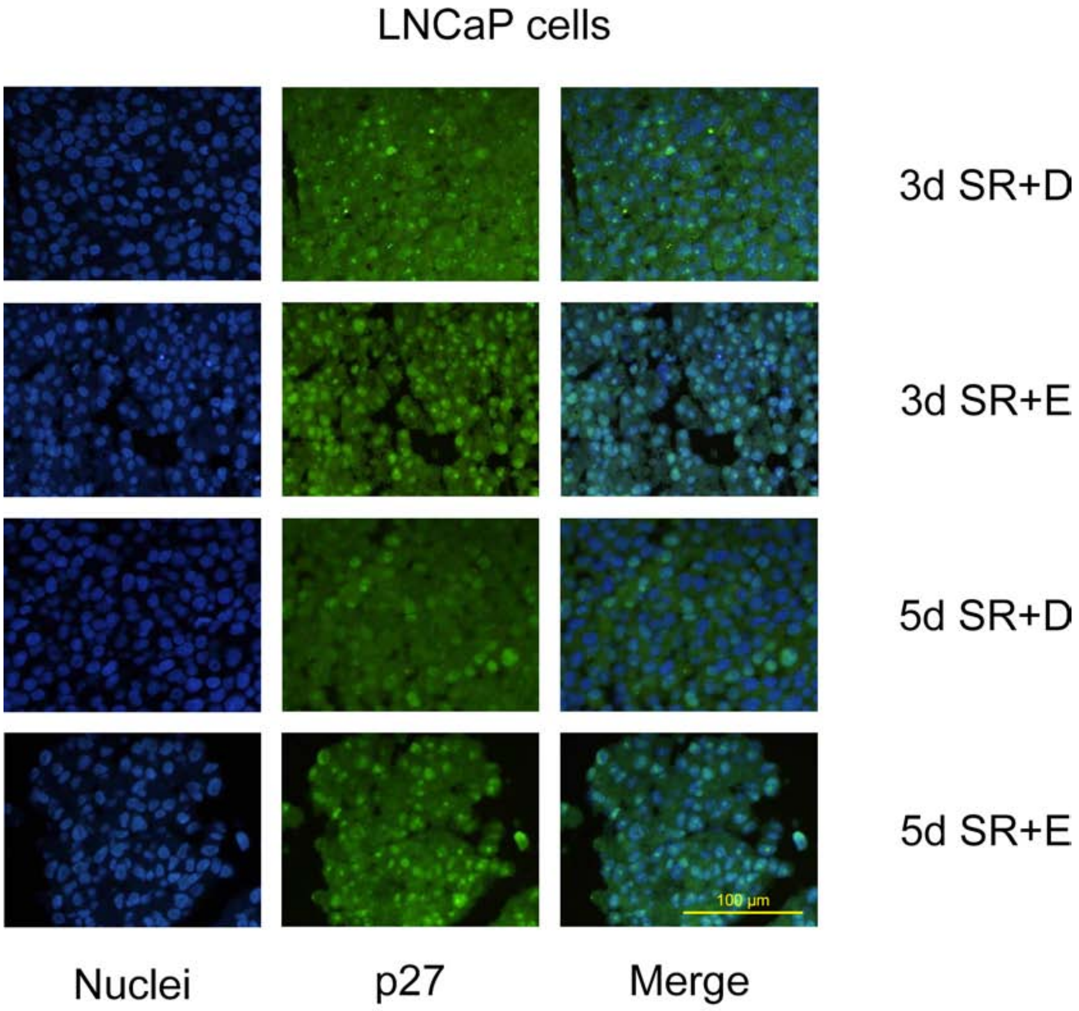

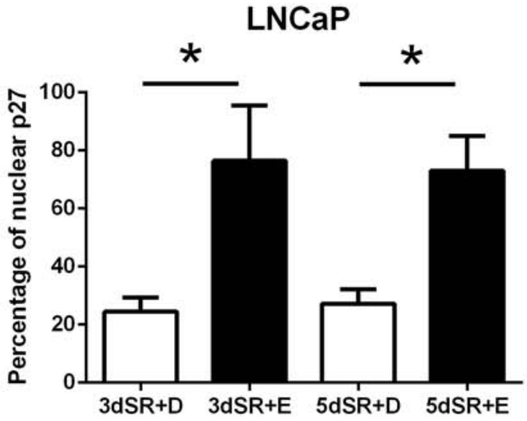

Supplementary Figure S7: Retention of nuclear p27 in LNCaP cells by Efipladib during cell cycle re-entry. Quiescent LNCaP cells were permitted to re-enter cell cycle by re-plating them the presence of serum in T75 flasks. Either 20 μM Efipladib or DMSO was administered upon serum restoration. Three days or five days after treatment, the cells harvested for immunofluorescence analysis of p27. The histograms show the percentage of nuclear positive cells and the data represent mean ± SD from one of three experiments. * Different to the vehicle control at each corresponding time interval ($p < 0.01$). 3dSR+D: treatment of serum-replenished (SR) cells with DMSO for 3 days. 3dSR+E: treatment of serum-replenished cells with Efipladib for 3 days. 5dSR+D: treatment of serum-replenished cells with DMSO for 5 days. 5dSR+E: treatment of serum-replenished cells with Efipladib for 5 days.
